# The role of volcanic-derived clays in the preservation of Ediacaran biota from the Itajaí Basin (ca. 563 Ma, Brazil)

**DOI:** 10.1038/s41598-021-84433-0

**Published:** 2021-03-03

**Authors:** Bruno Becker-Kerber, Abderrazak El Albani, Kurt Konhauser, Ahmed Abd Elmola, Claude Fontaine, Paulo S. G. Paim, Arnaud Mazurier, Gustavo M. E. M. Prado, Douglas Galante, Pedro B. Kerber, Ana L. Z. da Rosa, Thomas R. Fairchild, Alain Meunier, Mírian L. A. F. Pacheco

**Affiliations:** 1 Programa de Pós-Graduação em Ecologia e Recursos Naturais, Universidade Federal de São Carlos, Washington Luiz, 325km, São Carlos, SP 13565-905 Brazil; 2 Unité Mixte de Recherche (UMR), Centre National de la Recherche Scientifique (CNRS), IC2MP 7285, University of Poitiers, 86073 Poitiers, France; 3 University of Alberta, Edmonton, Canada; 4 Programa de Pós-Graduação em Geologia, Universidade do Vale do Rio dos Sinos, São Leopoldo, RS 93022-750 Brazil; 5 Instituto de Geociências, Universidade de São Paulo, Rua do Lago, 562, Cidade Universitária, São Paulo, SP 05508-080 Brazil; 6 Brazilian Synchrotron Light Laboratory (LNLS), Brazilian Center for Research in Energy and Materials, Av. Giuseppe Maximo Scolfaro, 10000, Campinas, CEP 13083-100 Brazil; 7 Instituto de Química, Universidade Federal de Mato Grosso do Sul, Cidade Universitária, Av. Costa e Silva-Pioneiros, Campo Grande, CEP 79070-900 Brazil; 8 Petrobras, Santos, SP Brazil; 9 Departamento de Biologia, Universidade Federal de São Carlos-Campus Sorocaba, Rod. João Leme dos Santos km 110, Sorocaba, CEP 18052-780 Brazil; 10 Instituto de Física, Universidade de São Paulo, Rua do Matão, Travessa R 187, São Paulo, CEP 05508-090 Brazil

**Keywords:** Planetary science, Geochemistry, Mineralogy, Palaeontology

## Abstract

The early evolution of metazoans has been reconstructed by studies on exceptionally preserved molds in siliciclastic rocks from the Ediacaran Period. However, there remains considerable controversy regarding the formation mechanisms of this unusual ‘Ediacaran-style’ preservation. Proposed hypotheses usually include early authigenesis of minerals, but evidence for this is scarce. In a recently discovered deposit of Ediacaran biota in Brazil, we show that the classic moldic preservation is related to clay mineral authigenesis. Specifically, these clays originated from the alteration of original pyroclastic sediments, likely enhanced by microbial activity, leading to early illitization and morphological templating of the fossiliferous surfaces at a micrometric scale. Such high-fidelity preservation was made possible by rapid burial during volcanic events and the in-situ templating of tissue by clays via microbially-mediated mineralization. This newly described *Lagerstätte* demonstrates that a number of minerals can facilitate preservation, and that perhaps ‘Ediacaran-style’ preservation result from different processes leading to the same broad style of preservation.

## Introduction

The typical moldic preservation of soft-bodied organisms in the Ediacaran Period (635–539 Ma)^1–4^, in particular the positive epirelief type where the fossils protrude up from bedding surfaces^4^, has few parallels in other periods of geologic time, hence the term “Ediacaran-style preservation.” One explanation points to rheological differences amongst the sediments^5^, a hypothesis that has received recent support^4^. Gehling^1^ also proposed the so-called ‘death-mask’ model for this preservation, in which microbial mats stabilized the sediments and produced a veneer of authigenic pyrite (FeS_2_) that replicated the morphology of the organisms. Evidence in favor of this hypothesis comes from the presence of iron oxide and pyrite concentrations along fossiliferous bedding planes in certain localities^3,6^. Other factors may include episodic event deposition^7^, early cementation by amorphous silica (SiO_2_)^2,8^, and burial by volcanic ash—the so-called “Conception-style” preservation^9^.

It is similarly possible that multiple mechanisms could produce this type of moldic fossilization and/or that taphonomic factors varied according to the geologic setting. In this regard, the Itajaí Basin^10–12^ (ca. 563 Ma, Brazil) appears to be a promising locality in which to study the possible mechanisms of preservation operating during the Ediacaran Period. This geological unit is relatively unmetamorphosed, has similar ages to the Avalon Assemblage, and also possesses high-fidelity preservation of the fossils. Recently, Becker-Kerber et al.^13^ described the Itajaí biota, reporting the presence of *Palaeopascichnus*, discoidal forms, micrometric filaments, Arumberia and fossilized microbial tufts. In this work, we apply a diverse array of techniques to address the fossilization processes of this new Ediacaran locality. More specifically, we demonstrate the close association of body fossils and microbial mats with volcanic sediments. We then provide a mechanism for the morphological preservation of tissue that includes the rapid formation of authigenic clays during early diagenesis as a result of the alteration of volcanic materials and microbial activity.

## Results

**Figure 1:**
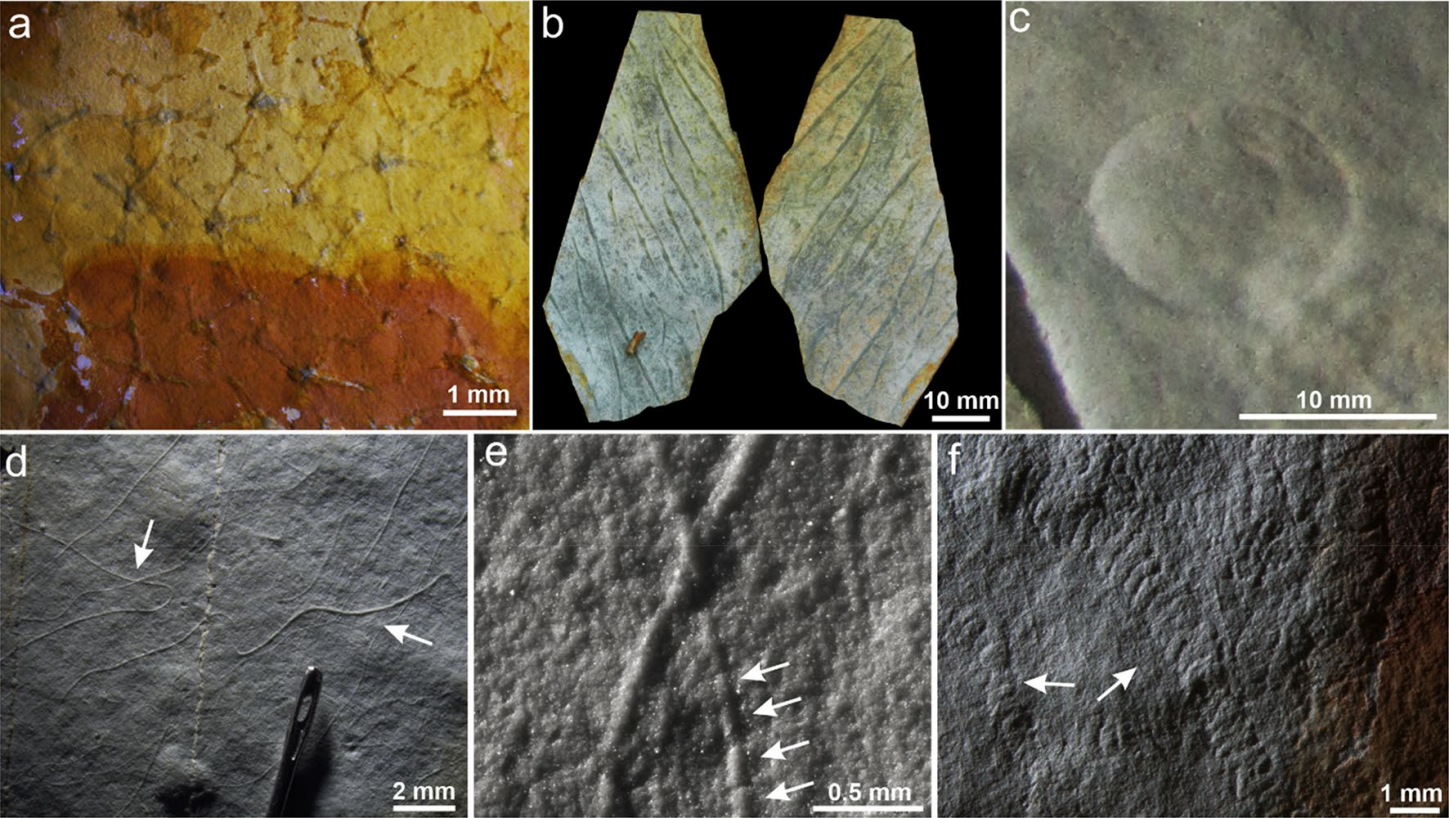
Ediacaran fossils and preservation styles in the Itajaí Basin. (**a**) Three-dimensionally preserved tufted microbial mats (CAP-879). (**b**) 3D Arumberia-type microbial mats (CAP-880). (**c**) *Aspidella* in positive hyporelief (CAP-549). (**d**) Sub-millimetric to micrometric filaments in positive epirelief (CAP-881). (**e**) Exquisite preservation of filaments bearing putative cellular structures (arrows) (CAP-866). (**f**) *Palaeopascichnus* in positive epirelief. Image created using Inkscape 1.0 (https://inkscape.org/).

**Figure 2:**
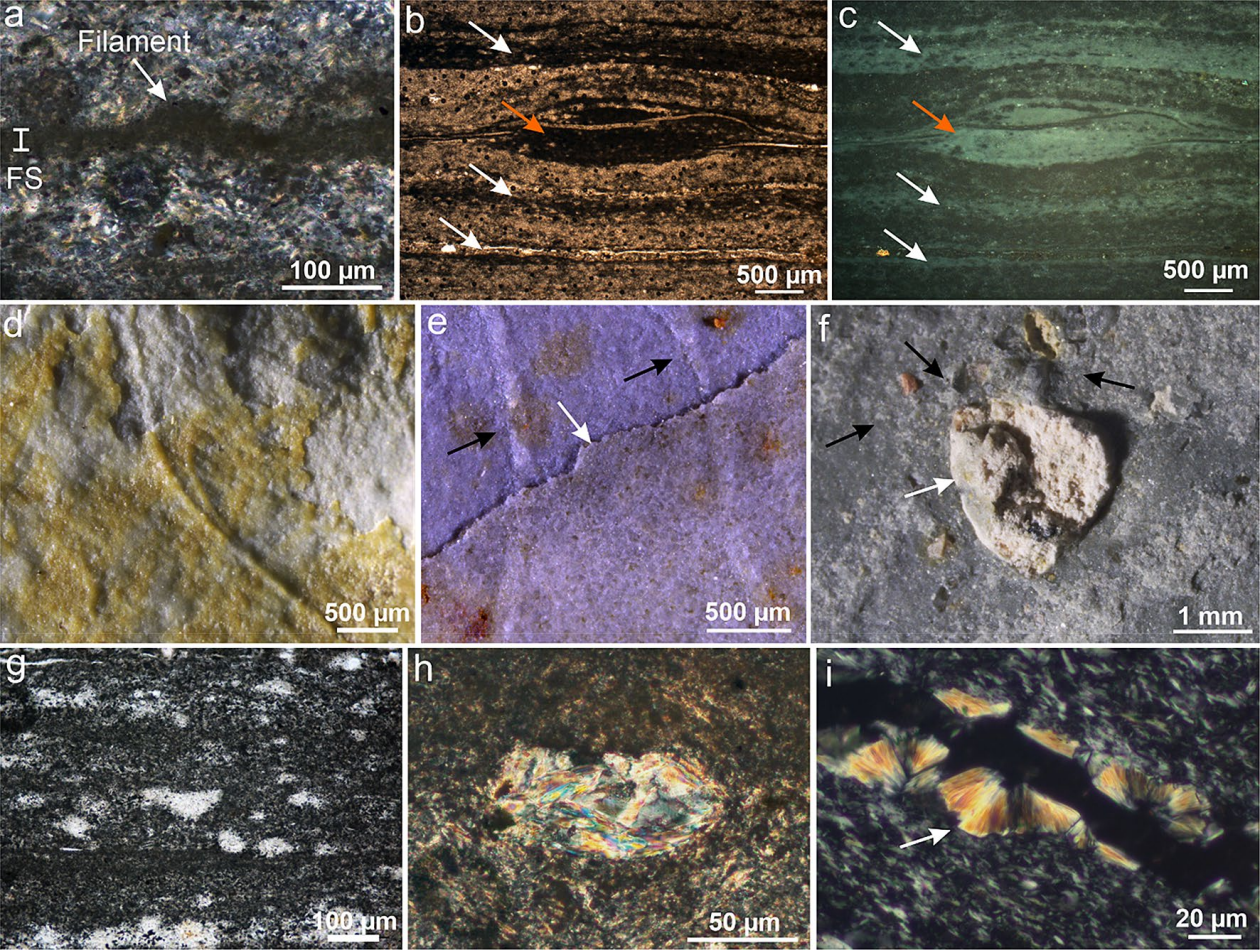
Pyroclasts from the fossiliferous horizons of the Itajaí Basin. (**a**) Vertical thin section through micron-sized filament. Note the concentration of authigenic clays on the fossiliferous surface (FS). Vertical thin section under transmitted (**b**) and reflected light (**c**) showing fossiliferous laminae (white arrows) and tuft (orange arrow) composed of authigenic clay minerals. (**d**, **e**) Micron-sized filaments covered by a layer of altered ash. (**f**) Accretionary pellet (white arrow) and scattered phenocrysts (black arrows). (**g**) Pyroclasts devitrified to microcrystalline quartz. (**h**) Angular clast devitrified to sericite. (**i**) Fibrous radial sericite in early-formed fractures. Note the molding relationship (arrow) with the host rock. Image created using Inkscape 1.0 (https://inkscape.org/).

The Itajaí biota is composed of the well-known chambered taxon *Palaeopascichnus*, the discoidal forms *Aspidella* and *Nimbia*, and micron-sized algal or bacterial filaments^13^ (Fig. 1c–f). Based on the mineralized width (33–193 µm) and bedding plane disposition of the filaments it is suggested that these could be the fossilized remnants of giant filamentous sulphur oxidizing bacteria or eukaryotic algae. Well-preserved reticulated and Arumberia-type microbial mats have also been observed (Fig. 1a,b). We detected three modes of preservation: (1) primarily impressions in positive epirelief (*Palaeopascichnus*, *Aspidella*, and micrometric filaments) (Fig. 1d–f); (2) rare impressions as negative epirelief (*Aspidella*) (Fig. 1c); and (3) full-relief, three-dimensional microbial mats replicated by clays (Fig. 1a,b). To our knowledge, this is the first case in the geological record of distinctive morphologies of microbial mats (e.g., reticulated tufts and Arumberia) being three-dimensionally replicated by clay minerals. Investigations by microtomography (µ-CT) corroborated the moldic preservation of the filaments (Supplementary Fig. S1a–c) as well as the unusual 3D nature of the microbial mats (Supplementary Fig. S1d–f, and Movie 1). Remarkably, very small filaments (c. 30 µm in width) are preserved as impressions, and some individuals seem to bear putative cellular structures (Fig. 1e). This minute detail indicates a level of morphological retention in molds that is unprecedented in the fossil record because filamentous microfossils are usually permineralized or occur as palynomorphs^14^.

All the preservation types mentioned above occur in the same lithology: pale-gray millimeter-scale rhythmites of mud and silt that were deposited in an upper slope setting subjected to rhyolitic volcaniclastic input (Supplementary Text 1, Supplementary Figs. S2, and S3). These layers additionally display abundant evidence of microbial mat development (e.g., reticulated mats, crinkly laminations; Fig. 1a, Supplementary Fig. S1d–f). The fossil impressions are located in authigenic (i.e., formed in situ) clay-rich laminations characterized by opaque and microcrystalline clay minerals (Fig. 2a; Supplementary Figs. S4 and S5). These surfaces can either occur between detrital clay (i.e., inherited) layers or at the bottom or top of siltstone laminae (Fig. 2a, Supplementary Fig. S5). The authigenic clays also compose the unique three-dimensional microbial mats at the same beds (Figs. 1a,b, 2b,c, Supplementary Figs. S4 and S6a). Identical textures are again observed in 3D clay-replicated microbial mats from distal delta-front millimetric-scale rhythmites (e.g., Arumberia; Supplementary Figs. S2 and S6b) associated with reworked volcaniclasts and positive epirelief impressions of *Nimbia*. By contrast, when microbially induced sedimentary structures (MISS) are found in non-volcaniclastic sediments, they are preserved only as impressions in semi-relief without associated authigenic minerals (see Ref.^13^).

The upper slope fossiliferous beds, which yielded the majority of samples, are represented by ash-fall pyroclasts, including elongate, cuspate, angular, and blocky devitrified vitric clasts; euhedral phenocrysts (some broken); accretionary pellets (sensu Brown et al.^15^); ash clusters; and coated particles (Fig. 2d–h, Supplementary Fig. S7a–f). Fine layers of altered ash were observed directly covering well-preserved micrometric filaments (Fig. 2d,e). Coarse-grained silt laminae interbedded with the fossiliferous horizons also contain volcaniclasts (Supplementary Fig. S4), which likely represent a mix of primary and reworked ash-fall deposits. These clasts exhibit devitrification features with the formation of clay minerals (Supplementary Fig. S7e,f), as well as clay rims and fluid and/or melt inclusions. Some tuffs near the fossiliferous levels also showed the presence of glass spheres (Supplementary Fig. S7g,h).

In thin section, the surfaces with fossil impressions are characterized by localized concentrations of very fine clays (Fig. 2a, Supplementary Fig. S5). In some layers, early-formed fractures developed mainly following these bedding surfaces. These fractures were filled by fibrous radial micaceous crystallites (sericite) and later by quartz. The formation of this fibrous sericite seems to have occurred before the lithification of the deposits given their molding relationship with the surrounding sediments (Fig. 2i). The early-filled fractures are observed in some instances to crosscut the clay-mineralized fossiliferous surfaces, implying an early origin for the fossil-bearing clays.

X-ray diffraction (XRD) patterns and mid-infrared (MIR) and near-infrared (NIR) spectra further reveal that the fossil-bearing clays (clay-rich surfaces with fossil impressions and clay-replicated microbial mats; hereafter FBCs) are mineralogically similar to the clays from the volcanic sediments (volcaniclastic laminae—VL, tuffs, and tuffites), but differ from other facies throughout the basin that do not show abundant volcaniclasts or clay mineralized fossils (see Supplementary Text 2, Supplementary Figs. S8–S18, Dataset S1). Both FBCs and clays from the volcanic-derived sediments show a predominance of illite (1 M polytype) and ordered illite–smectite mixed-layer minerals (R3 I–S MLMs) as suggested by the shoulder on the left side of the illite peak (at 10 Å) and further confirmed by NEWMOD modelling (Supplementary Figs. S12 and S13).

**Figure 3:**
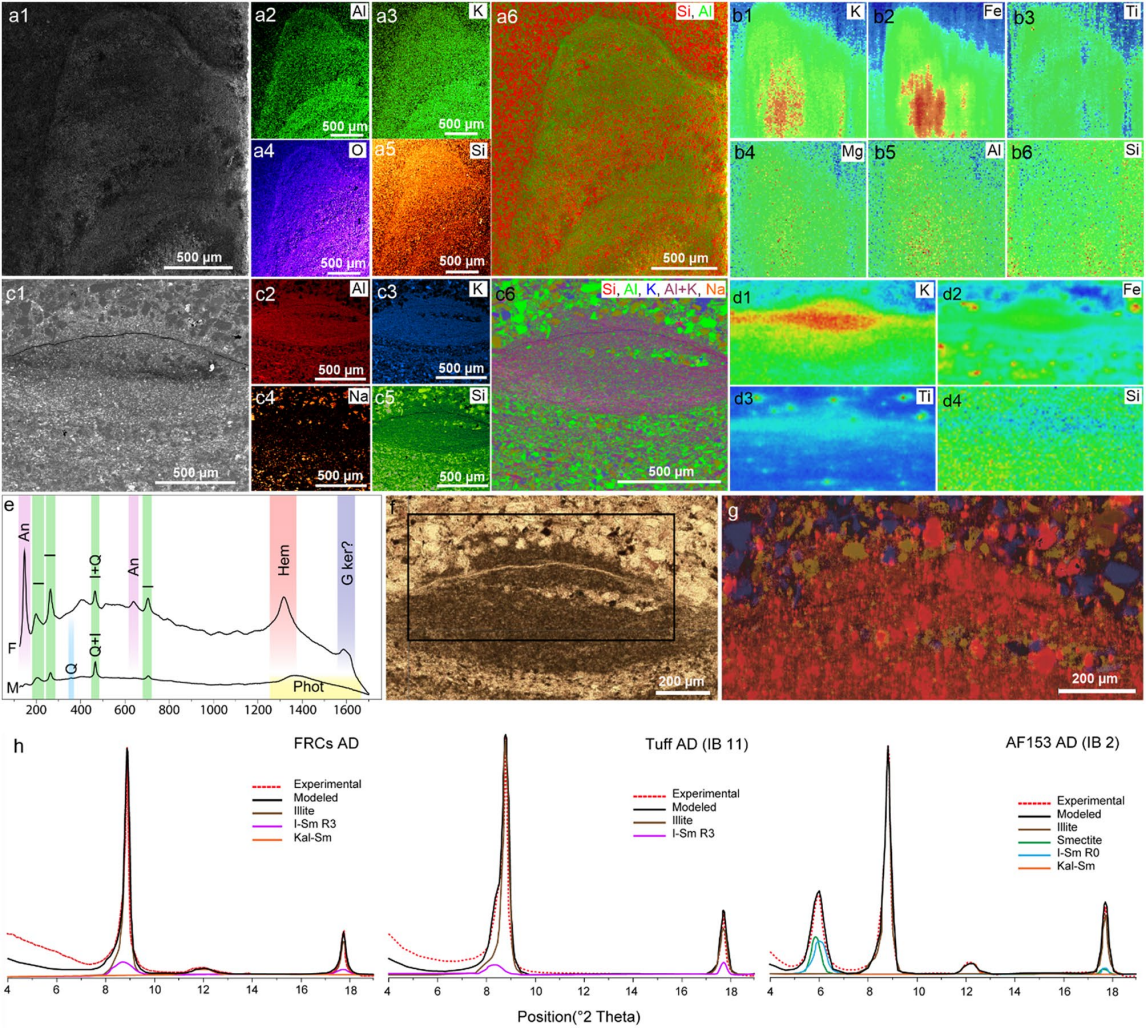
Geochemical analyses of the fossil-related clays (FBCs) in three-dimensionally preserved microbial mats. (**a**) SEM image and EDS maps of a region diagonally crosscutting a 3D preserved tufted microbial mat (see Supplementary Fig. S6), highlighting the concentration of Al and K in the biogenic structure. (**b**) SR-µXRF maps of the same region, showing increased intensities of K, Fe, Ti, Mg and Al in the fossil-bearing clays. (**c**) SEM image and EDS maps of a cross-section through a 3D preserved Arumberia-type microbial mat (see Supplementary Fig. S6), revealing relatively higher intensities of Al and K in the fossil. (**d**) SR-µXRF of the same region as in (**c**), showing enrichments and spatial relations of K, Fe and Ti with the microbial mat. (**e**) Representative Raman spectra of FBCs (F) and rock matrix (M). *An* anatase, *I* illite, *Q* quartz, *Hem* disordered hematite (see Ref.^46^), *G ker* possible G band of kerogen, *Phot* artifact of photoluminescence from the glass. (**f**, **g**) Raman mapping of anatase (red), quartz (blue) and plagioclase (yellow) of the Arumberia sample. Note the concentration of disseminated and fine-grained anatase in the fossil. (**h**) XRD patterns (air-dried) and NEWMOD modeling of the FBCs, the tuff sample, and a distal delta-front mudstone sample. Figures created using INCA (**a**, **c**; https://www.etas.com/en/products/inca_software_products.php), PyMCA 5.4.0 (**b**, **d**; http://pymca.sourceforge.net/), WiRE (**e**–**g**; https://www.renishaw.com/) and NEWMOD 2.0 (**h**) software.

As a comparison, the clay assemblages from facies with no fossil-bearing clays are represented by smectite and randomly ordered R0 I/S MLMs (Fig. 3h, Supplementary Text 2, and Supplementary Figs. S8–S18), as well as kaolinite/smectite and Chl-S MLMs that originated during weathering and late diagenetic processes, respectively (Supplementary Text 2). Moreover, the prevalence of the 2 M polytype suggests that most of the illite/muscovite from these samples are detrital in origin.

At higher resolution, the XRD data show that the FBCs exhibit a higher proportion of illite than the clays from the adjacent VL of the same beds, and the tuffs and tuffites (Fig. 3h, Supplementary Figs. S11 and S12). SEM micrographs illustrate this difference by evidencing the crystal habit of illite for the clays inside the 3D replicated microbial mats, while the crystal habits of I–S MLMs dominated the host rock (Supplementary Fig. S19).

Scanning electron microscopy with energy dispersive X-ray spectrometry (SEM–EDS) and synchrotron radiation micro-X-ray fluorescence spectroscopy (SR-µXRF) confirmed the higher K concentration and the illite composition of the FBCs. Indeed, these analyses showed that the FBCs have high concentrations of K, Al, Fe, Ti, and Mg (Fig. 3a–d), while the host rock contains high concentrations of elements related to the nature of the clasts and cements (e.g., Si, Na, and Fe). Their illite composition was further confirmed by the oxide weight% results of EDS point analyses (Dataset S2) showing the following average composition: (Si_3.56_, Al_0.44_)O_10_(Al_1.63_, Mg_0.11_, Fe_0.20_)(OH)_2_(K_0.61_, Na_0.01_). Finally, Raman spectroscopy of the 3D clay-replicated microbial mats additionally reveals the characteristic peaks of illite (ca. 200 cm^−1^, 264 cm^−1^, 396 cm^−1^, 464 cm^−1^, and 703 cm^−1^) (Fig. 3e, Dataset S3). The results from the fitting of several spectra demonstrate that this illite is indeed authigenic in origin and more concentrated in the FBCs than the cement/matrix of the adjacent host rock (Supplementary Text 3, Supplementary Fig. S20).

Raman spectroscopy reveals that anatase (TiO_2_) is the Ti-bearing phase in the 3D clay-replicated microbial mats (Fig. 3e–g, Dataset S4). This anatase is formed of micrometer- to submicrometer-sized crystals (Fig. 3g, Supplementary Fig. S21). Anatase is especially concentrated in the microbial mats, while the matrix contains a high proportion of another TiO_2_ mineral, rutile (Supplementary Text 3, Dataset S3). The greater concentration of anatase may explain the dark appearance of the clay-rich fossiliferous surfaces and 3D replicated mats under petrographic observation.

## Discussion and conclusion

Our results show that the studied fossil impressions are preserved in surfaces composed of authigenic illite and are associated with diverse volcanic sediments (Figs. 2, 3). In the case of the microbial mats, they are even three-dimensionally replicated by the authigenic clays (Figs. 2b,c, 3a–g). Indeed, the fossiliferous horizons and volcanic sediments are enriched in authigenic illite when compared to other levels throughout the basin (Supplementary Text 2, Supplementary Figs. S8–S18). Despite that illite/muscovite is also a component of the clay assemblage at other (non-fossiliferous) levels (e.g., Supplementary Figs. S8–S13), it likely reflects a detrital origin given the practical absence of the 1 M polytype and the predominance of the 2 M polytype.

Illite is usually the result of the progressive alteration of smectite during diagenesis depending on temperature, time, and K^+^ availability^16^. Therefore, the concentration of authigenic illite in the fossil horizons and volcanic materials suggest the original presence of authigenic smectite or illite–smectite clay minerals. Furthermore, the absence of prograde diagenesis (Supplementary Text 2; Supplementary Figs. S8–S10) shows that the higher illitization of the FBCs and volcanic sediments are unrelated to the diagenetic maturation of the rocks and more likely represent K^+^ availability from the volcanic sediments.

Importantly, the mineralogical similarity and the direct association of volcanic material with the fossil-bearing clays suggest that the volcanic sediments played a major role in the formation of these authigenic clays. In fact, the alteration of unstable volcanic glass is well known to promote the formation of new silicate phases^17–24^. Their instability is due to nonbridging silicate sites, which allow easy alteration at low temperatures^18^. In this regard, both experimental and field-based studies have shown the formation of smectite and R0 I–S MLMs from the alteration of volcanic glass occurring at low temperature^19–22^.

The abundant evidence for the presence of microbial communities at the fossiliferous beds (Figs. 1a, 2b,c; Supplementary Figs. S4, and S5; see also Ref.^13^) supports the idea that the alteration of volcanic sediments could have been enhanced by microbial activity. For instance, Kawano and Tomita^23^ demonstrated that bacterially influenced alteration of pyroclastic sediments can lead to smectite formation through an allophane precursor, while Konhauser et al.^24^ showed the formation of smectite directly on the cell surfaces of bacteria colonizing basaltic tephra.

Moreover, the higher amount of illite in the R3 I–S MLMs from the FBCs than in the adjacent VL and tuffs (Fig. 3; Supplementary Figs. S10, and S11) advocates a link between the presence/activity of microbial communities and processes of early diagenetic illitization, which could have influenced fossil preservation. Two mechanisms are envisaged to explain this higher illitization: (1) further enrichment in K^+^ resulting from microbial metabolism and/or (2) dissimilatory iron reduction (DIR) of Fe^3+^ in smectite. The former hypothesis is similar to the one proposed for the microbial mats of the Paleoproterozoic Francevillian Group^25^, where the presence and/or metabolism of ancient microbial mats served as the source of K^+^ for the illitization of these microbially related surfaces. Although K-feldspar grains from the volcanic sediments in the Itajaí Basin likely contributed to posterior diagenetic illitization, their presence does not account for the higher illitization of the FBCs than the clays in the millimeter apart volcaniclastic laminae, as well as the tuffs levels throughout the basin (Fig. 3h, Supplementary Figs. S10, and S11).

The second mechanism (DIR) is related to the dissolution of smectite layers during the illitization process. This process could also account for the presence of disordered hematite in the FBCs (see Kim^26^). Furthermore, Zhang et al.^27^ demonstrated that illitization by DIR can be enhanced by an external supply of Al and K, which in the Itajaí Basin could have come from volcaniclastic material. The release of dissolved iron may have also contributed to the adsorption of this element by the surfaces of algal or bacterial cells—including both the cell walls and/or extracellular polymeric substances (EPS)—as observed in modern examples^28^. This process can subsequently result in the formation of sites prone to the nucleation of clay mineral phases^28–30^. Several other studies have documented the role of microbes in providing sites for the nucleation and growth of clay and silicate phases^31–34^.

In addition to the higher degree of illitization, the enrichment in anatase (Fig. 3e–g, Supplementary Fig. S21), as well as disordered hematite (Fe_2_O_3_—Fig. 3e), inside the clay-replicated microbial mats further supports the influence of microbial metabolism in the microenvironmental conditions at the authigenic clay surfaces. Studies have shown that Fe and Ti can be released in the microenvironment through the bioleaching of ilmenite (FeTiO_3_) by Fe(II)-oxidizing and/or iron-scavenging bacteria (i.e., those that produce siderophores)^35^, thus creating conditions for the precipitation of new phases, such as anatase. Furthermore, experiments with photosynthetic microbial mats have shown the influence of biological activity on the precipitation of anatase when ilmenite was added to the medium^36,37^. In the Itajaí Basin, Fe–Ti oxide grains (e.g., ilmenite and titanomagnetite—now altered to leucoxene; Supplementary Fig. S22) are common in the volcaniclastic sediments and are a likely source of anatase and Fe enrichment in the ancient mats.

**Figure 4:**
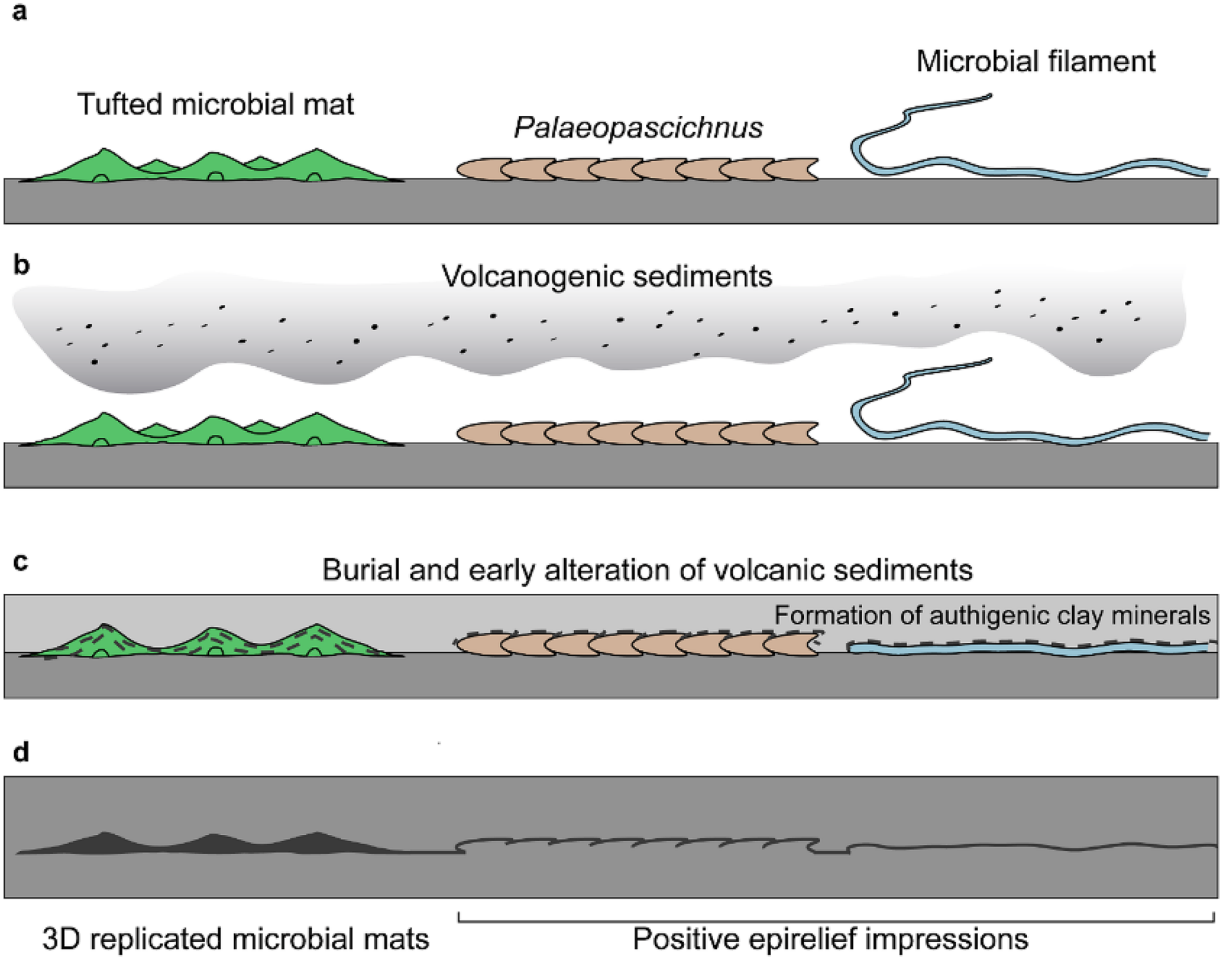
Schematic representation of the fossilization pathway for the Itajaí Biota. (**a**) Living organisms at the sediment–water interface. (**b**) Deposition of volcanogenic sediments (reworked and/or ash fall). (**c**) Early diagenesis and alteration of volcanic sediment to clay minerals. (**d**) Authigenic clays preserving the external surfaces of the fossils and replicating the three-dimensional morphology of the microbial mats.

**Figure 5:**
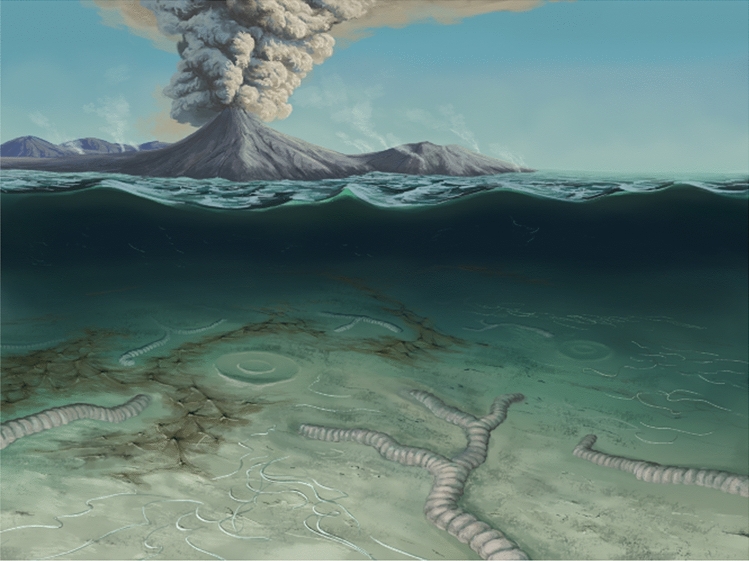
Artistic representation of the volcanic and microbial influences on the preservation of Itajaí organisms. Artistic reconstruction by J. S. d’Oliveira.

According to our results, in the FBCs and volcanic sediments of the Itajaí Basin, smectite and/or I–S MLMs were likely the primary mineral phases derived from the alteration of volcanic sediments. This process, supported by microbial activity, resulted in microenvironments enriched in cations, such as K^+^, Al^3+^, and Na^+^, and then to the precipitation of newly formed clay minerals. Therefore, we hypothesize that a combination of abundant and easily-altered volcanic particles—in addition to microbial activity—were the essential conditions necessary for the preservation of the Itajaí biota. This complex interplay between volcanic sediments and microbial activity culminated in the formation of abundant early diagenetic clays (Figs. 4, 5), resulting in the 3D substitution of microbial constructions and in templating the fossil-bearing surfaces with clay precipitates, thus preserving the external morphologies of macro-organisms and micron-sized algal or bacterial filaments. Besides, the early precipitation of clays may have also helped in the cementation and stabilization and of the sediments.

The role of volcanic material in the preservation of certain Ediacaran assemblages has been previously proposed for the conception-style preservation^9^. However, details explaining this mechanism have not been provided to support this hypothesis, and until now, no authigenic minerals have been associated with this model. Moreover, recent studies have even extended the death-mask model to these deposits, where pyritization would have played a major role in the moldic fossilization^3^. Our work shows that, at least for some basins, the fossilization of the Ediacaran macroscopic communities was the result of the interconnected processes related to volcanic and microbial activity (Figs. 4, 5).

Volcanic activity has often been considered one of the main causes of biotic events during the history of life on Earth, and here we demonstrated unequivocally that volcanism also played an important role in preserving the information of ancient ecosystems. Other examples are also known from the fossil record^38–45^ and show that volcanism was not only important for exceptional preservation throughout the geologic record but also diverse in terms of processes and composition of the precursor material.

## Materials and methods

### Macroscopic and petrographic investigation

Macroscopic features of hand samples were analyzed using a Stereomicroscope ZEISS SteREO Discovery V20 coupled with an Axiocam camera at the Laboratório de Ecologia at the Universidade Federal de Mato Grosso do Sul. The preparation of thin sections was carried out in the Programa de Pós-Graduação em Geologia da Universidade do Vale do Rio dos Sinos (UNISINOS—São Leopoldo). Approximately 119 thin sections of the fossiliferous levels, tuffites, tuffs, and other facies were prepared to compare the petrographic data with the mineralogical results from the other techniques. Representative thin sections were polished for a better characterization of the volcaniclasts and devitrification features. We used Zeiss microscopes at the UNINOS and Université de Poitiers (France).

### X-ray diffraction and NEWMOD modeling

Whole-rock powder and clay mineral fractions (< 2 µm) from 24 samples were analyzed with a Panalytical Xpert Pro diffractometer at the University of Poitiers using Cu (Kα = 1.541874 Å) radiation, with the following configuration: Xccelerator detector; geometry θ/θ (Bragg–Brentano); goniometer of 240 mm radius; wavelength filter of Ni (0.3 mm thick); anti-divergence slit of 1/8°2θ, anti-diffusion slit of ¼°2θ; a mask of 10 mm in diameter; soller slit of 0.04 rd spacing; and fixed sample holder. The analyses were performed with a voltage of 40 kV and a current of 40 mA in the angular range of 2–65°2θ (powder) and from 2 to 35º 2θ (oriented preparation).

Twenty-two samples originated from different levels (fossiliferous and nonfossiliferous) throughout the basin, while the fossil clays and the intercalated coarse-grained laminae were carefully selected and removed from the fossiliferous beds. These fossiliferous samples were first prepared using air abrasive processes to grossly clean and remove the surrounding matrix from the 3D clay-mineralized microbial mats. Then, we extracted the clays from the selected structures and laminae (fossilized microbial mats and intercalated coarse-grained laminae) under a stereomicroscope using a scalpel. All samples were ground in a mortar and separated for powder analysis. Later, we extracted the clay size fraction (< 2 µm) for oriented preparation. These clay size fractions were then Ca saturated and mounted on glass slides for analysis in air-dried ethylene glycol and after thermal treatment (i.e., 350 °C and 550 °C).

For the characterization of the I–S MLMs, we used NEWMOD simulation to model the Reichweite ordering parameter (R), which can range from randomly interstratified (R = 0) to long-range ordered MLMs (R = 1, 2, and 3).

### Scanning electron microscopy

We used a Quanta 650FEG and FEI Inspect F50 microscopes (project SEM-21836 and SEM-23684) at the Brazilian National Laboratory of Nanotechnology (LNNano/CNPEM) and a JEOL JSM IT500 scanning electron microscope, equipped with secondary electron, backscatter electron detectors and coupled with a Bruker lynxeye Energy Dispersive X-ray Spectrometer (EDX) at the IC2MP laboratory of the Université de Poitiers. Analyses were conducted in high-vacuum mode and with a current tension of 15 kV. Quantitative SEM point analyses were performed for clay minerals and the corresponding structural formulas were calculated from the total oxides weight percent.

### SR-μXRF

The SR-μXRF investigation was performed at the Brazilian Synchrotron Light Laboratory (LNLS) under proposals 20,171,031 and 20,180,327. We used polychromatic excitation in microbeam mode and filtering with Fe foils for the measurements. For the FlyScan mode, we applied 500 ms of count time per point. For data treatment, we used the PyMCA 5.4.0 software.

### Raman spectroscopy

We used a Renishaw InVia microRaman with 785 nm lasers and 17 mW total power (attenuated). The spectra acquisition for all the points in the clays was performed using the same configuration: 785 nm laser; 1 s of exposure time; 100 accumulations; laser power of 50%; static acquisition with a center in 750; and objective of 100×. This approach yielded robust data for the statistical comparisons of the fossil clays, matrix, and detrital micas. These spectra were obtained in two different time periods and thus compared separately regarding peak position since small variations can occur in the equipment. Both groups of data presented the same pattern of results (see Supplementary Fig. S20). The Raman spectra of the Ti-oxides were realized using the same configuration, except for laser power (5% in this case). Baseline subtraction and fitting were performed using WiRE 4.1. Raman maps were obtained using the streamline method.

### Mid-infrared and near-infrared (MIR, NIR)

We used the clay fractions of the samples for the MIR and NIR investigation. For the MIR, we prepared KBr discs with 1 mg of sample and 149 mg of KBr. The mixture was ground and then pressed under 8 tons for 5 min to create the discs. Then, the samples were analyzed by a Nicolet iS50 Fourier transform infrared spectrometer using a DTGS KBr detector with a resolution of 4 cm^−1^ and an accumulation of 100 scans in the range of 4000–400 cm^−1^. The NIR was performed in a Nicolet 6700 Fourier transform infrared spectrometer. All spectra were processed using the OMNIC 9.9.473 software (www.thermoscientific.com/pm_molspec).

### X-ray microtomography (µ-CT)

The X-ray microtomographic analysis was performed at the PLATINA platform of the IC2MP (University of Poitiers) with the RX-Solutions EasyTom XL Duo device. We used a microfocus X-ray source (Hamamatsu L8121-03) coupled with a flat panel imager (Varian PaxScan 2520 DX). The acquisition parameters were as follows: 120 kV voltage, 200 µA current, 1 mm Al filter, stack acquisition with 2880 projections in two turns to acquire the whole sample, eight frames per second, an average of ten frames per projection, with anti-ring shift procedure and a spatial resolution of 18.3 µm. The data reconstructions were computed using XAct (RX Solutions) and virtual sections: 3D rendering and movies were produced using Avizo v.2019.2 (Thermo Fisher-FEI). X-ray microtomography was also performed at the Brazilian Synchrotron Light Laboratory (LNLS) in the IMX beamline in the energy range of 5 keV to 20 keV with an average flux of 8.1 × 1013 photons/s/mm^2^ using the white beam. We used a Si filter of 350 µm. The sample was rotated in 180°, and the time of exposition varied according to the intensity of the transmitted beam. The images were reconstructed with the pyRaft62 algorithm. We then processed the reconstructed images using the software Amira 6.2 and Avizo 9.1.

## Supplementary Information

Supplementary Information 1.

Supplementary Information 2.

Supplementary Information 3.

Supplementary Information 4.

Supplementary Information 5.

Supplementary Video S1.
