# Supplementary material for: The role of volcanic-derived clays in the preservation of Ediacaran biota from the Itajaí Basin (ca. 563 Ma, Brazil)
Source: Sci Rep. 2021 Mar 3;11:5013. doi: 10.1038/s41598-021-84433-0 (PMC7930025; doi:10.1038/s41598-021-84433-0)
Supplement: Supplementary file 1 — Supplementary Information 1. [file 41598_2021_84433_MOESM1_ESM.docx]

**The role of volcanic-derived clays in the preservation of Ediacaran biota from the Itajaí Basin (ca. 563 Ma, Brazil)**

Bruno Becker-Kerber^a,b*^, Abderrazak E. Albani^b^, Kurt Konhauser^c^, Ahmed A. Elmola^b^, Claude Fontaine^b^, Paulo S. G. Paim^d^, Arnaud Mazurier^b^, Gustavo M.E.M. Prado^e^, Douglas Galante^f^, Pedro B. Kerber^g^, Ana L. Z. da Rosa^h^, Thomas R. Fairchild^e^, Alain Meunier^b^, Mírian L. A. F. Pacheco^i^

Supplementary Text 1 – Geologic setting

The Itajaí Group is interpreted as a foreland basin composed of four or five depositional sequences with occasional volcaniclastic input ^1–5^ (DS1–5) (Supplementary Fig. S2, and S3). However, there remains controversies regarding the stratigraphic position of the possible DS 5, and for that reason it was excluded from our generalized stratigraphic column (Supplementary Fig. S2). Some authors interpret the DS5 deposits as a repetition of the previous units due to overthrusting. Zircon radiometric dating of tuff levels of the DS1 revealed sedimentation ages of 563± 3 Ma ^6^, agreeing with previous reported ranges for the deposition of the Itajaí Basin (572–549 Ma; see ref ^5^)

At the base, the DS 1 is characterized by alluvial deposits grading to deltaic-shallow marine settings. The overlying DS2 is composed by turbidite deposits, and upsection by upper-slope sediments. DS3 is defined mainly by shallow marine and prodelta deposits. DS4 is also characterized by shallow marine settings, but in this case with more influence of fluvial and proximal delta sediments. The possible DS5 repeats the facies observed in the previous sequences, being composed of turbidites and deltaic deposits. Further discussion on the stratigraphy, depositional environments and tectonic setting of the basin can be found in Rostirolla ^3^, Rostirolla et al. ^1^, Gresse et al. ^7^, Fonseca ^8^, Teixeira et al. ^9^, Guadagnin ^5^, Basei et al. ^4^. Here we follow the approach of Becker-Kerber et al. ^6^ in adapting the depositional sequences defined by Fonseca ^8^ and Teixeira et al. ^9^.

**Supplementary Text 2 – Clay assemblage of the Itajaí Basin**

Clay minerals are the most abundant minerals in sedimentary rocks, representing as much as 40% of the minerals in these rocks^10^. They can be found in geological systems as: (1) detrital/inherited; when they are transported by weathering agents and accumulate in other places different from the initial parent rocks; and/or (2) authigenic/newly formed; when they form in-situ and observed in their initial parent rocks. We observed both detrital and authigenic clay minerals in the Itajaí Basin, and we distinguished between them based on the morphology, texture, polytypes, and chemistry.

XRD patterns, MIR and NIR spectra of distinct facies from the Itajaí Basin show that the content of smectite and R0 illite–smectite mixed layer minerals (I–S MLMs) increases towards shallower and proximal settings of the delta deposits, while fossiliferous horizons display similarities with tuffs and tuffites (Supplementary Figs. S8–S18, Dataset S1). The similarities of fossiliferous beds and volcanic sediments (i.e., the higher illite content) in the IR absorption bands are clearer in the Si-O-Si stretching region near 1030 cm^-1^, in the OH stretching band near 3622 cm^-1^ and in the 2v(OH) overtone in the NIR region near 7072 cm^-1^ (Supplementary Figs. S15, S16, and S18). It is interesting to note also that the absorption bands related to the presence of Mg in octahedral sheets seems to be more intense in the fossiliferous levels and volcanogenic sediments (Supplementary Fig. S17), corroborating the data from the elemental analyses (main text).

Interestingly, in the MIR region between 3000 and 2800 cm^-1^, spectra from both the fossil-bearing clays (FBCs) and the volcanic material show higher intensities in the absorption bands related to the CH_3_ and CH_2_ stretching vibrations of aliphatic compounds than those found in samples without fossil-bearing authigenic clays (Supplementary Fig. S16). This finding reinforces the interpretation that volcanic sediments played a role in improving the preservation of the remaining organic material.

The smectite enrichment in proximal facies likely reflects river-borne sediments, similar to those observed in modern cases ^11^. Conversely, the random occurrence of kaolinite–smectite MLMs (Supplementary Figs. S8–S18), as well as its disordered and iron-rich nature (Supplementary S15, S16, and S18), suggests a later formation stage by acidic alteration of original clay minerals and/or other minerals (e.g., feldspars, micas), as seen in thin sections (data not shown). Interestingly, the turbidite complex of the depositional sequence 2 (DS2) also showed an increase in smectite content (Supplementary Fig. S9), which likely reflects the reworking of the previous deltaic deposits. Chlorite–smectite MLMs are also present in some samples and its concentration increases in localities closer to intruding rhyolite bodies or to the basin border (e.g., AF236, AF153, AF222, AF09, AF172; Supplementary Fig. S8–10). This suggests that this Chl–S MLMs results from the later alteration of smectite-rich rocks near these zones.

In sum, the results show a complex clay assemblage for the Itajaí Basin, related to sedimentological and diagenetic processes. Importantly, we corroborate the genetic association of the authigenic clays in the fossils and in the volcanic sediments, ruling out other hypotheses for clay enrichment in the fossiliferous surfaces and structures.

**Supplementary Text 3 – Raman spectroscopy results**

Statistical comparisons of the values obtained by the fitting of the Raman point analyses (Dataset S3) showed that bands of illite are more prevalent in FBCs than in the matrix/cement. Despite that the spectra from the matrix/cement also showed illite, the higher presence of quartz is evident by the common appearance of the weak quartz band near 355 cm^-1^ (Dataset S3). The concentration of clay minerals in the fossils is further evidenced by the higher ratios of 700 cm^-1^/464 cm^-1^ peak area (Supplementary Fig. S20c). Higher full width at half maximum (FWHM) values and lower positions of the peak near 464 cm^-1^ also differentiate the fossils from the matrix (Supplementary Fig. S20a, b). These features are related to the higher influence of the illite band near 464 cm^-1^, instead of the one representing quartz (Supplementary Fig. S20a, b). When compared to detrital micas, the peak at 700 cm^-1^ from both the FBCs and matrix clays show similar positions and higher FWHM variation (Supplementary Fig. S20d, e), reflecting not only different mineralogy from the detrital micas, but also a less crystallized clay mineral of authigenic origin.

Raman point analyses in randomly chosen TiO_2_ crystals showed a higher proportion of anatase (ca. 147 cm^-1^, 399 cm^-1^, 639 cm^-1^) (compared to rutile) in the FBCs (87.9%; n = 66) than the host rock (66.1%; n = 65) (Dataset S4). Moreover, the point analyses in the clay minerals demonstrated the elevated presence of disseminated fine-grained anatase (Fig. 3e) in the fossils (98.8%, n = 81) compared to the points in the matrix/cement (39.8%, n = 93) (Dataset S3, and S4).


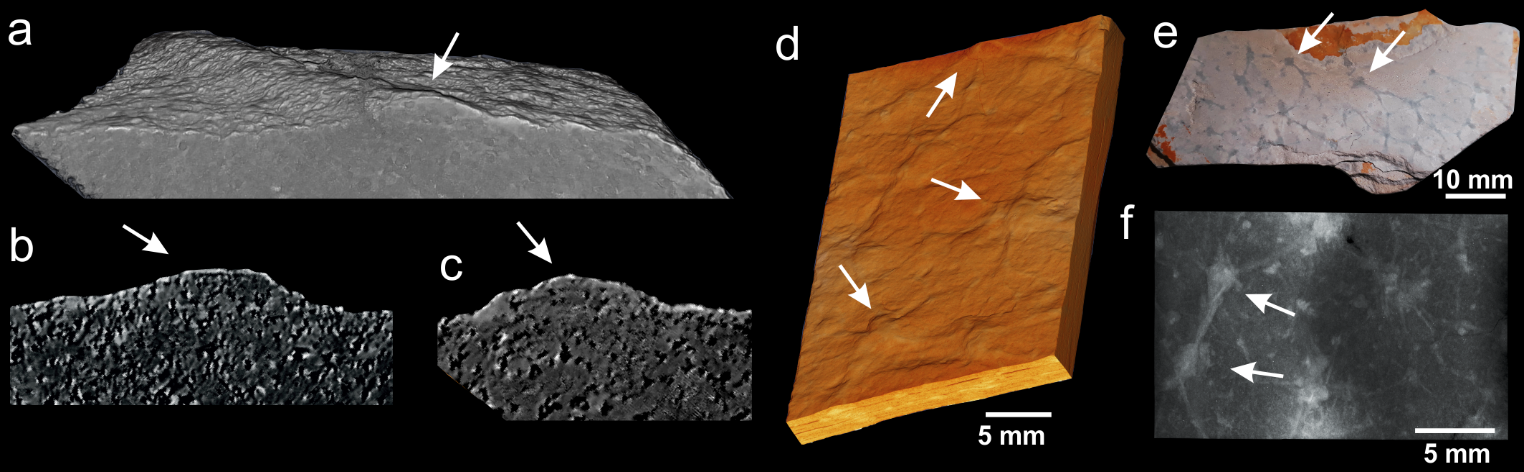


**Supplementary Figure S1**. **µCT of microbial filaments and 3D microbial tufts**. (**a)** 3D reconstruction of microbial filaments and sectional views. (**b-c)** The same clastic texture in the fossils as in the matrix. (**d)** 3D view of the surface of a sample containing preserved tufts that can be distinguished from the surrounding rock in polished (**e**) and microtomographic sections (**f**). Images created using Amira 6.2 and Avizo 9.1 (https://www.thermofisher.com/).


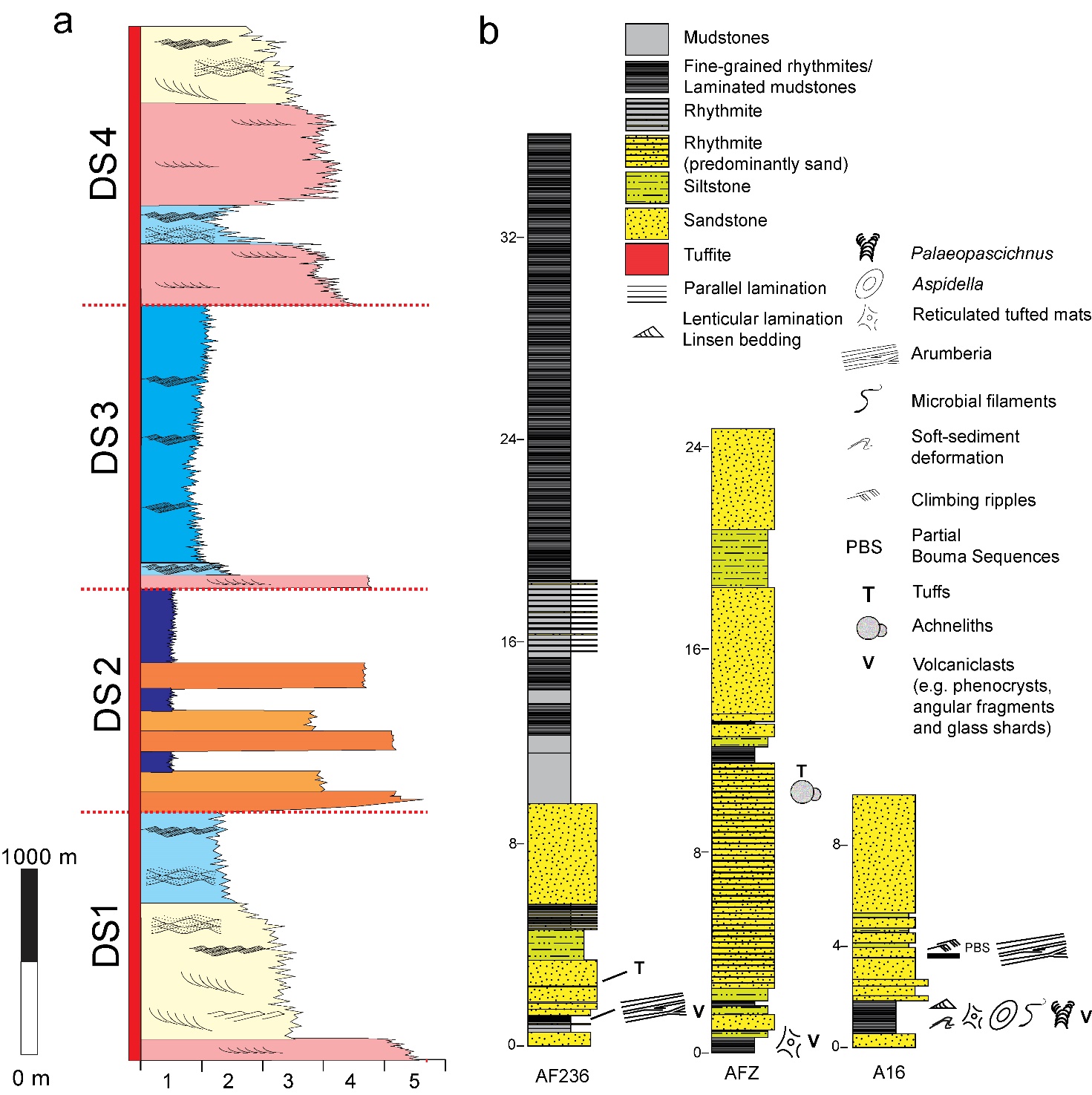


**Supplementary Figure S2**. **Stratigraphic framework of the Itajaí Basin**. **(a)** A generalized stratigraphic column of the basin, with the main depositional sequences: DS1; DS2; DS3; and DS4. Depositional Sequence 5 was omitted due to its uncertain stratigraphic relationship. **(b)** Stratigraphic logs of the main fossiliferous outcrops: AF236; AFZ; and A16.


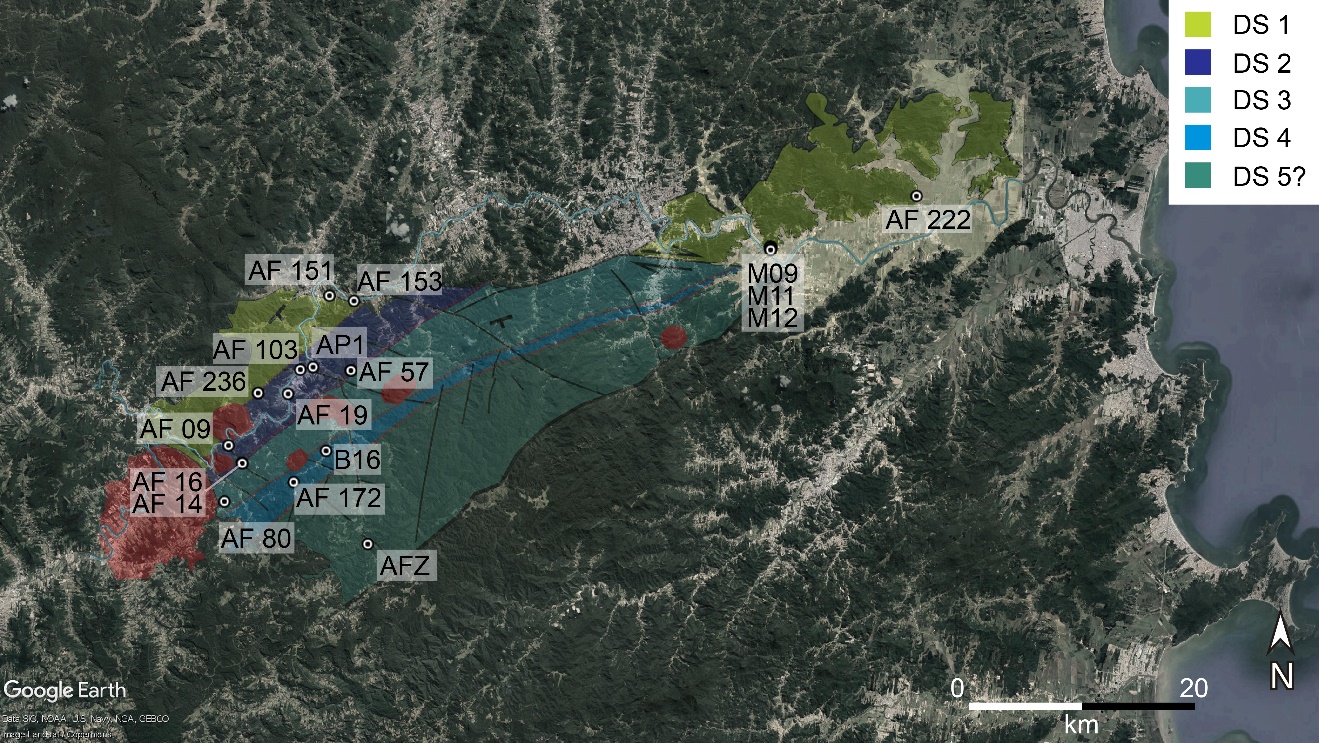


**Supplementary Figure S3**. **Geographic localization of the outcrops investigated in this study.** The depositional sequences occur from northwestern to southeastern as from base to top, being: DS1, DS2, DS3, DS4, and DS5. Satellite image obtained from Google Earth Pro 7.3.3.


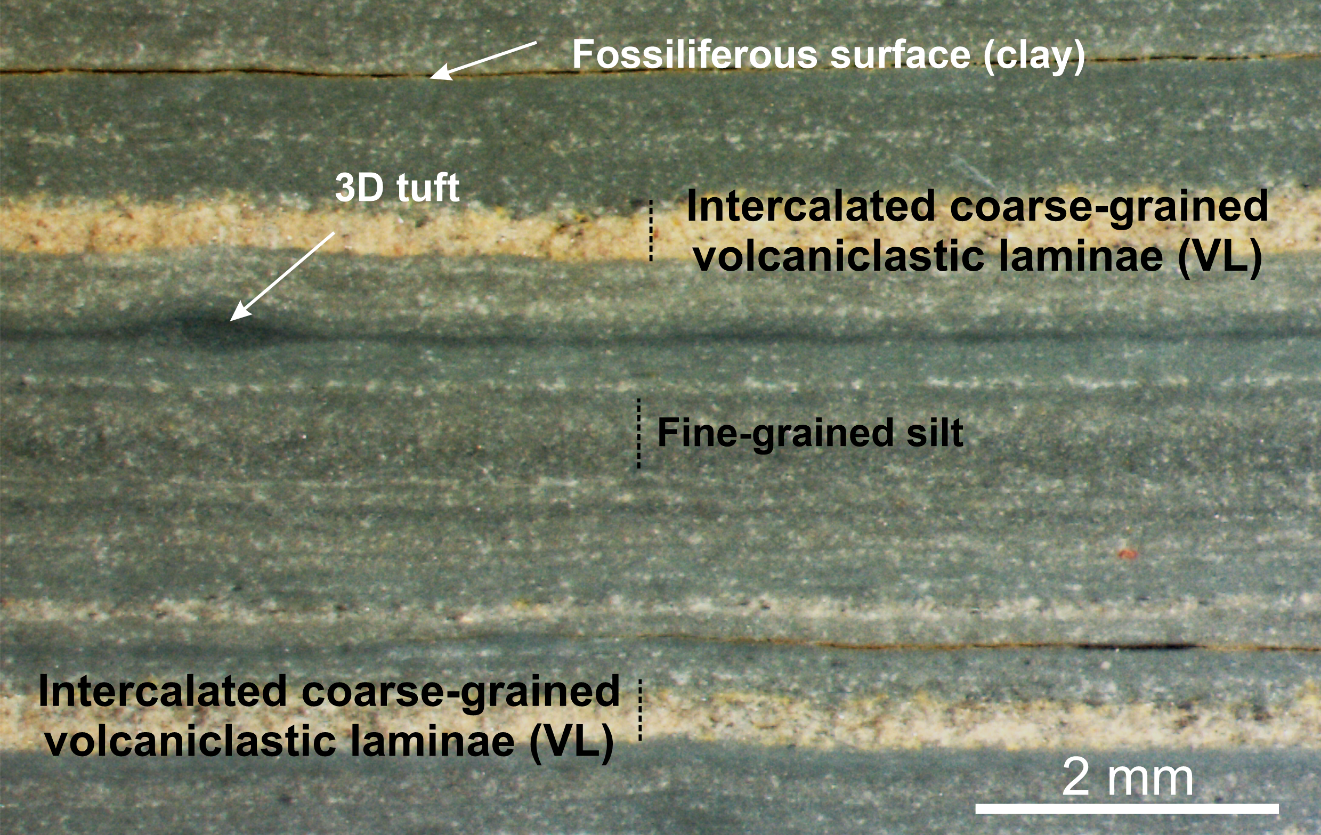


**Supplementary Figure S4**. **Sedimentary laminae.** Polished cross-section of a hand sample showing the millimeter-scale laminations comprising clay, fine-grained silt and coarse-grained volcaniclastic silt (VL), and the clays preserving the three-dimensional microbial mats.


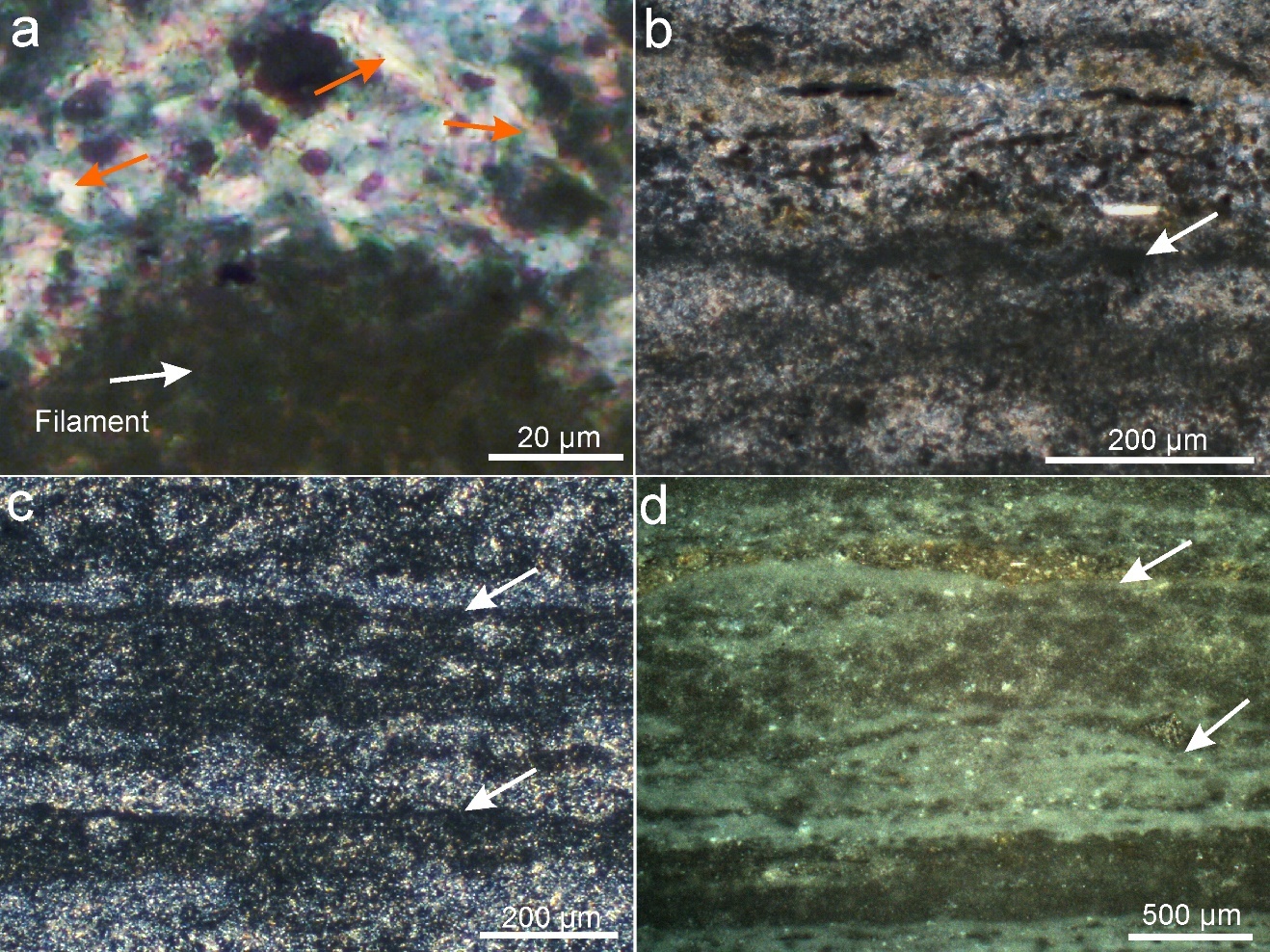


**Supplementary Figure S5**. Fossiliferous surfaces enriched in authigenic clay minerals. **(a)** Close-up view of the Fig. 2a of the main text, showing details of a filament in vertical section (white arrow) and the above sedimentary laminae rich in detrital phyllosilicates (orange arrows). **(b–d)** Further examples of fossiliferous surfaces with abundant authigenic clays, which appear dark in thin sections and whitish in reflected light (d).


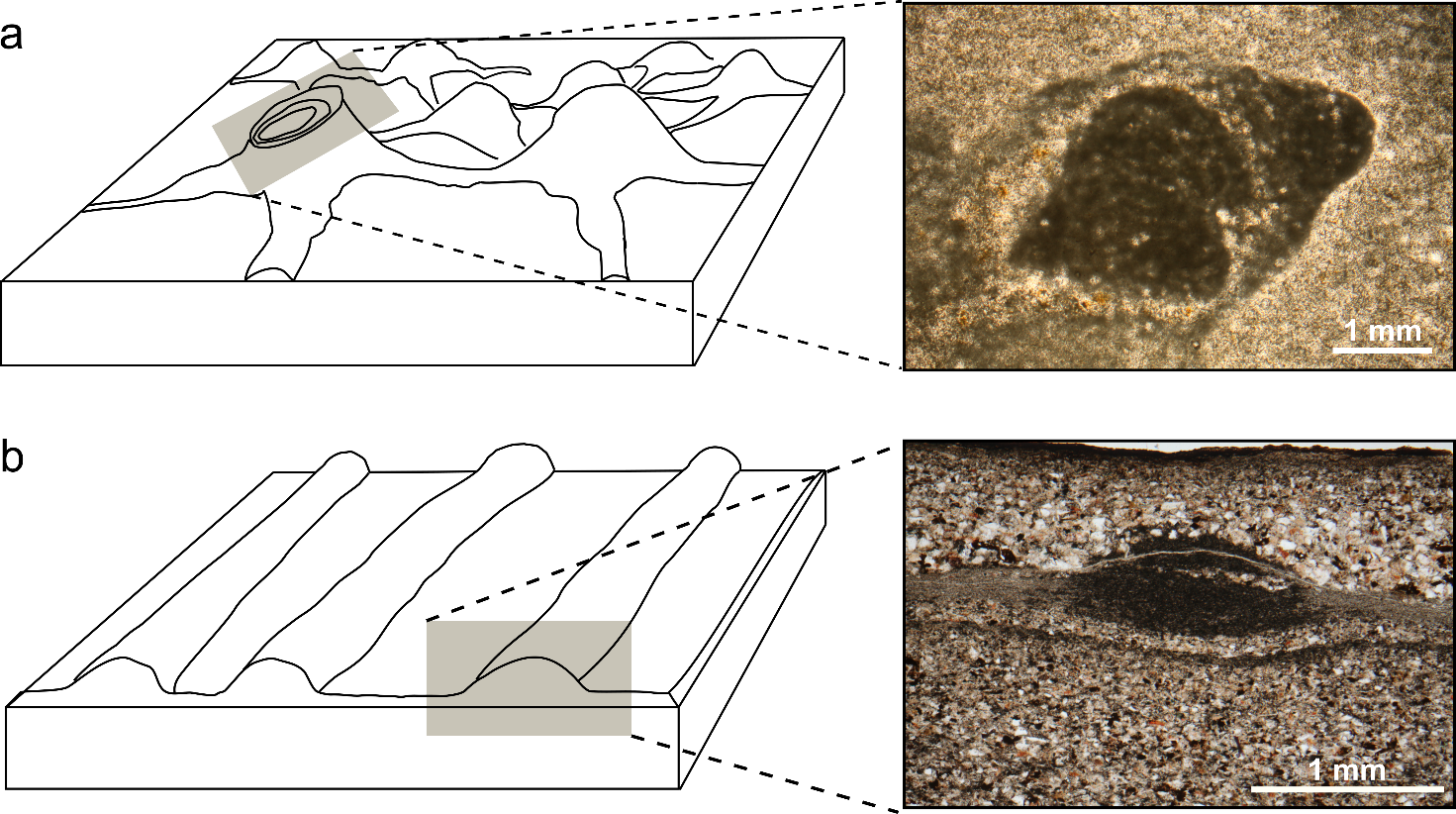


**Supplementary Figure S6. Three-dimensionally preserved microbial** **mats. (a)** Reticulated mats. **(b)** Arumberia-like mats in vertical section. Insets showing the specific orientations of the thin sections figured to the right. **Image created using Inkscape 1.0 (https://inkscape.org/).**

**
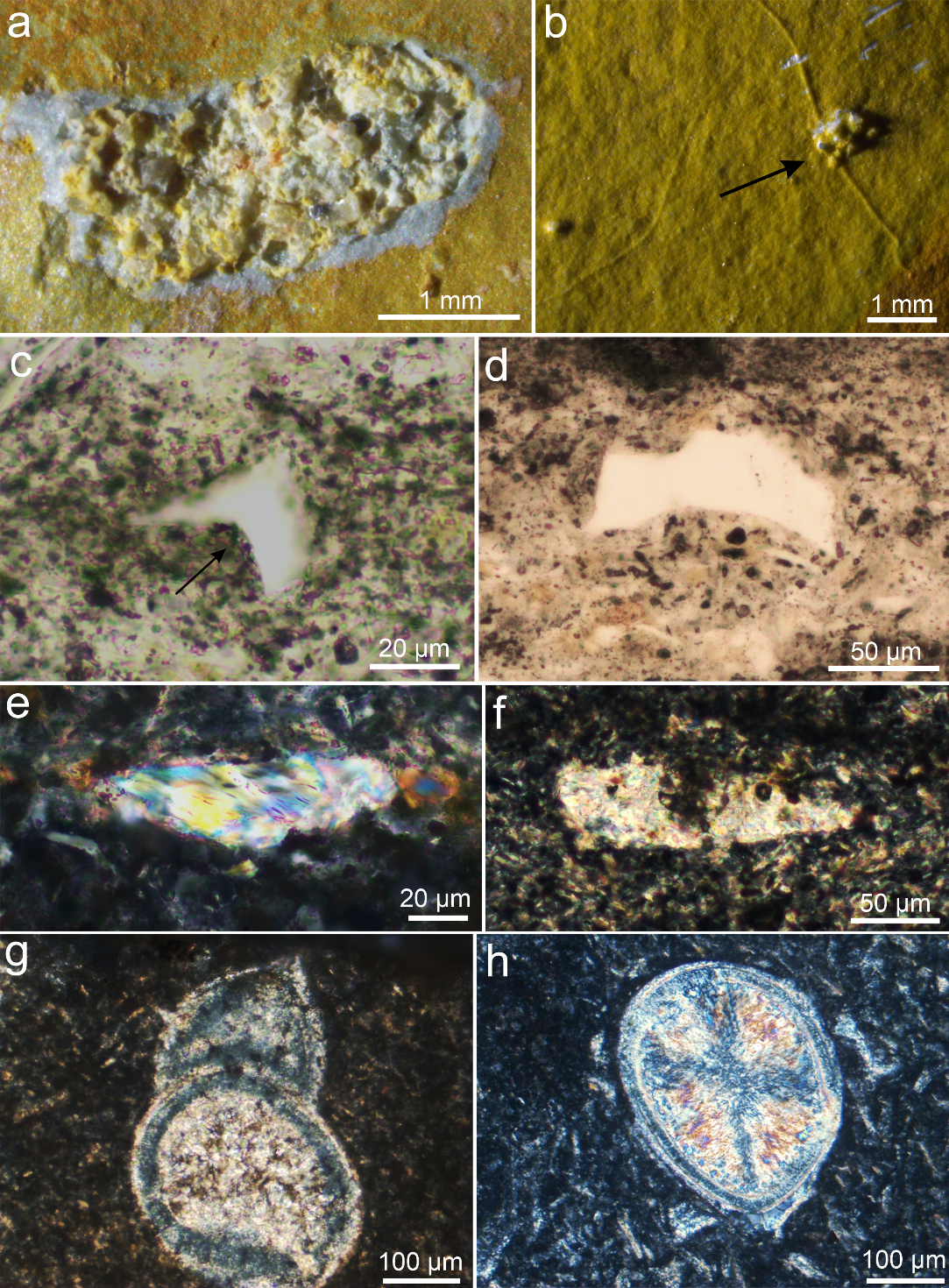
**

**Supplementary Figure S7. Accretionary pellets, pyroclasts/volcaniclasts, and glass spheres. (a)** Slightly reworked accretionary pellets composed of euhedral pyroclasts. **(b)** Ash cluster associate with fossils. **(c, d)** Vitric clasts and possible glass shards found associated with the fossiliferous surfaces. **(e, f)** Devitrified glassy clasts with angular morphologies**. (g, h)** Glass spheres in cherty tuffites.


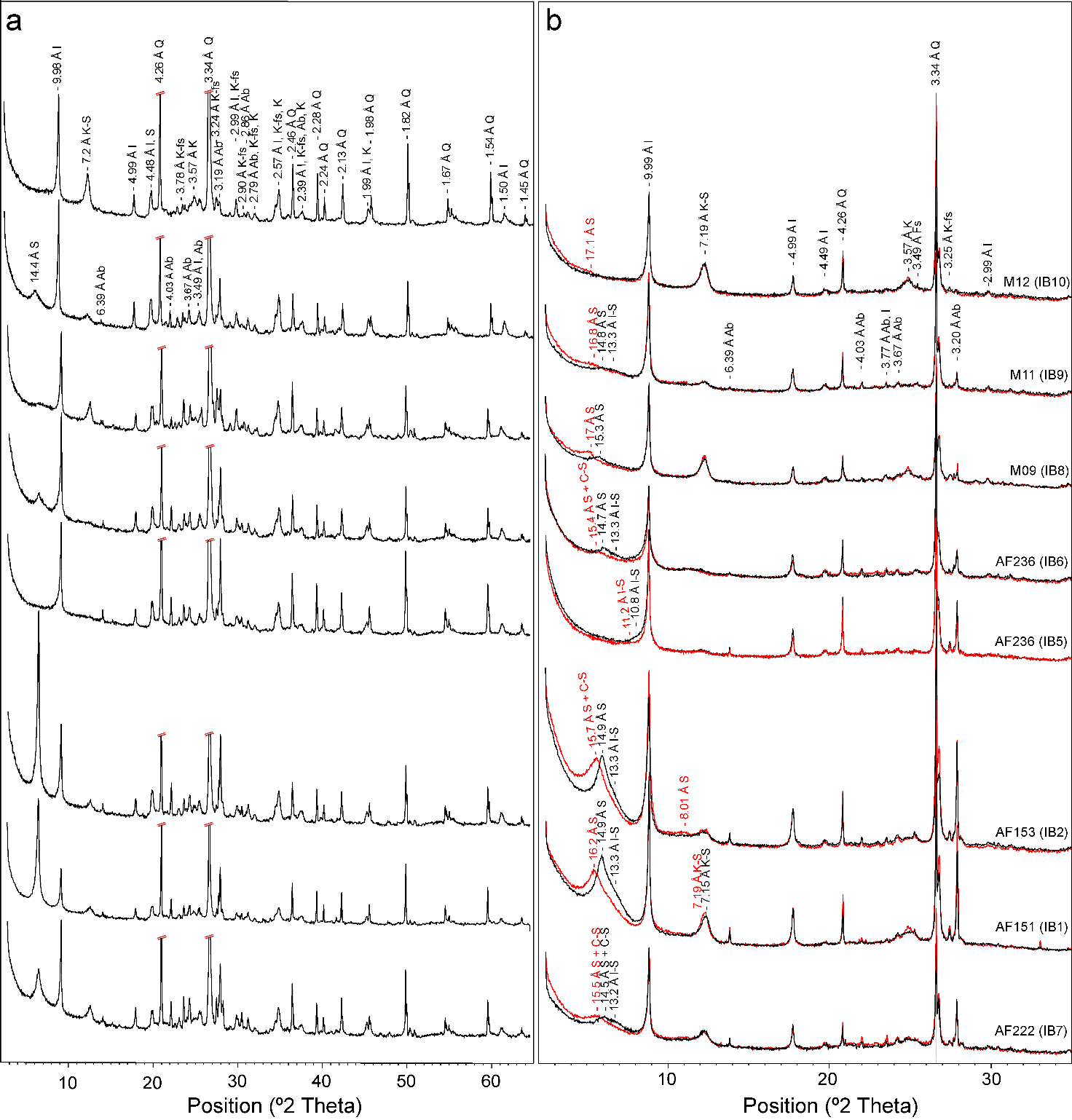


**Supplementary Figure S8. XRD patterns of representative samples from the depositional sequence 1.** Graphs are organized by their relative stratigraphic position in the DS1. **(a)** Bulk powder and **(b)** clay size-fractions (<2 µm). Note the higher content of smectite and/or smectite-rich I–S MLMs in the lower sections (more proximal depositional settings). I – Illite; S – Smectite; Q – Quartz; K-fs – K-feldspar; Ab – Albite; K – Kaolinite; I-S – Illite-Smectite mixed layer; C-S – Chlorite-Smectite mixed layer; K-S – Kaolinite-Smectite mixed layer.


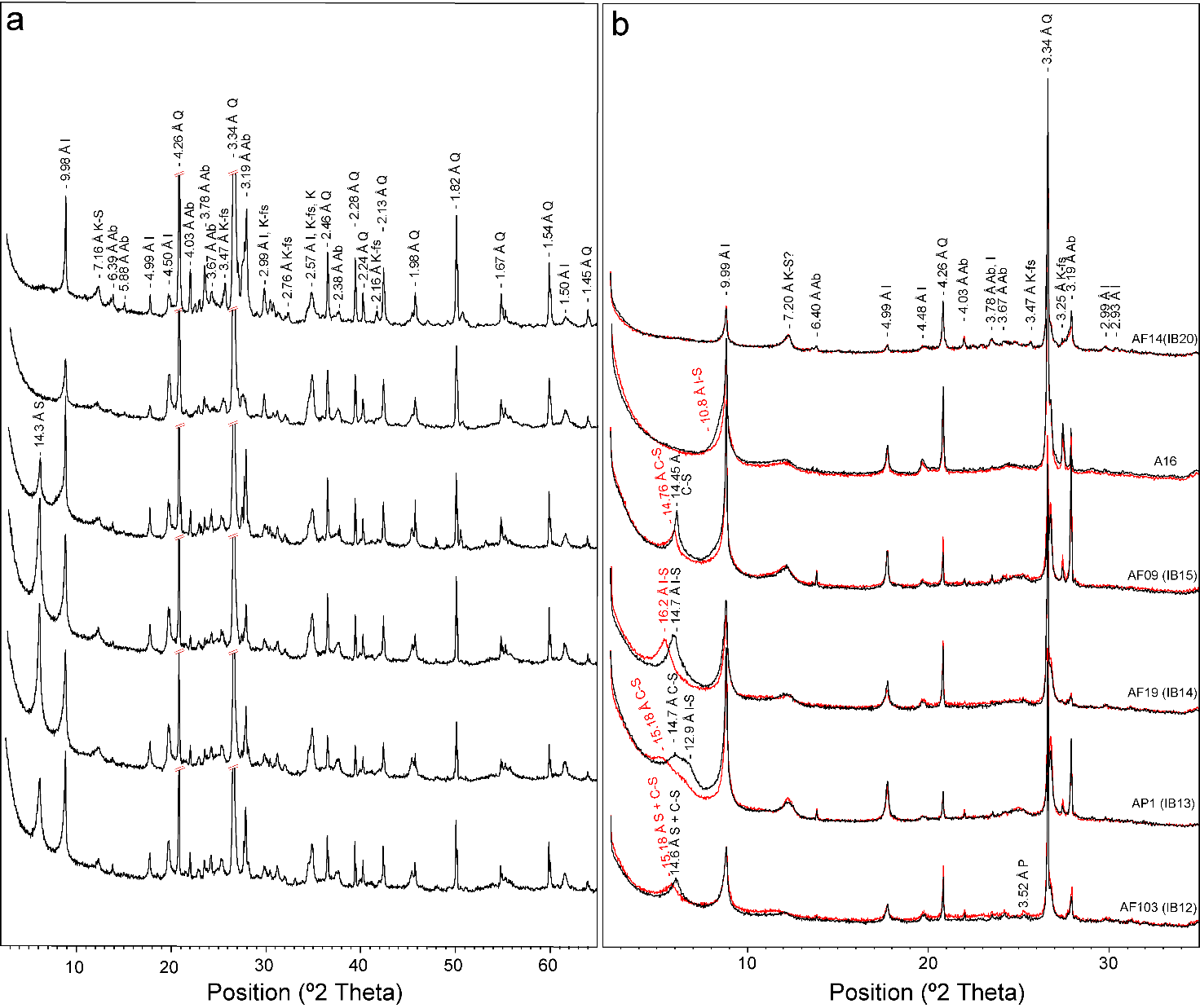


**Supplementary Figure S9. XRD patterns of representative samples from the depositional sequence 2.** Graphs are organized by their relative stratigraphic position in the DS2. (**a)** bulk powder and **(b)** clay fractions (< 2 µm). Note the higher smectitic content in the turbiditic complex of the DS2 (IB 12–15). Sample A16, which represents the fossiliferous outcrop, presenting R3 I–S MLMs. I – Illite; S – Smectite; Q – Quartz; K-fs – K-feldspar; Ab – Albite; K – Kaolinite; I-S – Illite-Smectite mixed layer; C-S – Chlorite-Smectite mixed layer; K-S – Kaolinite-Smectite mixed layer.


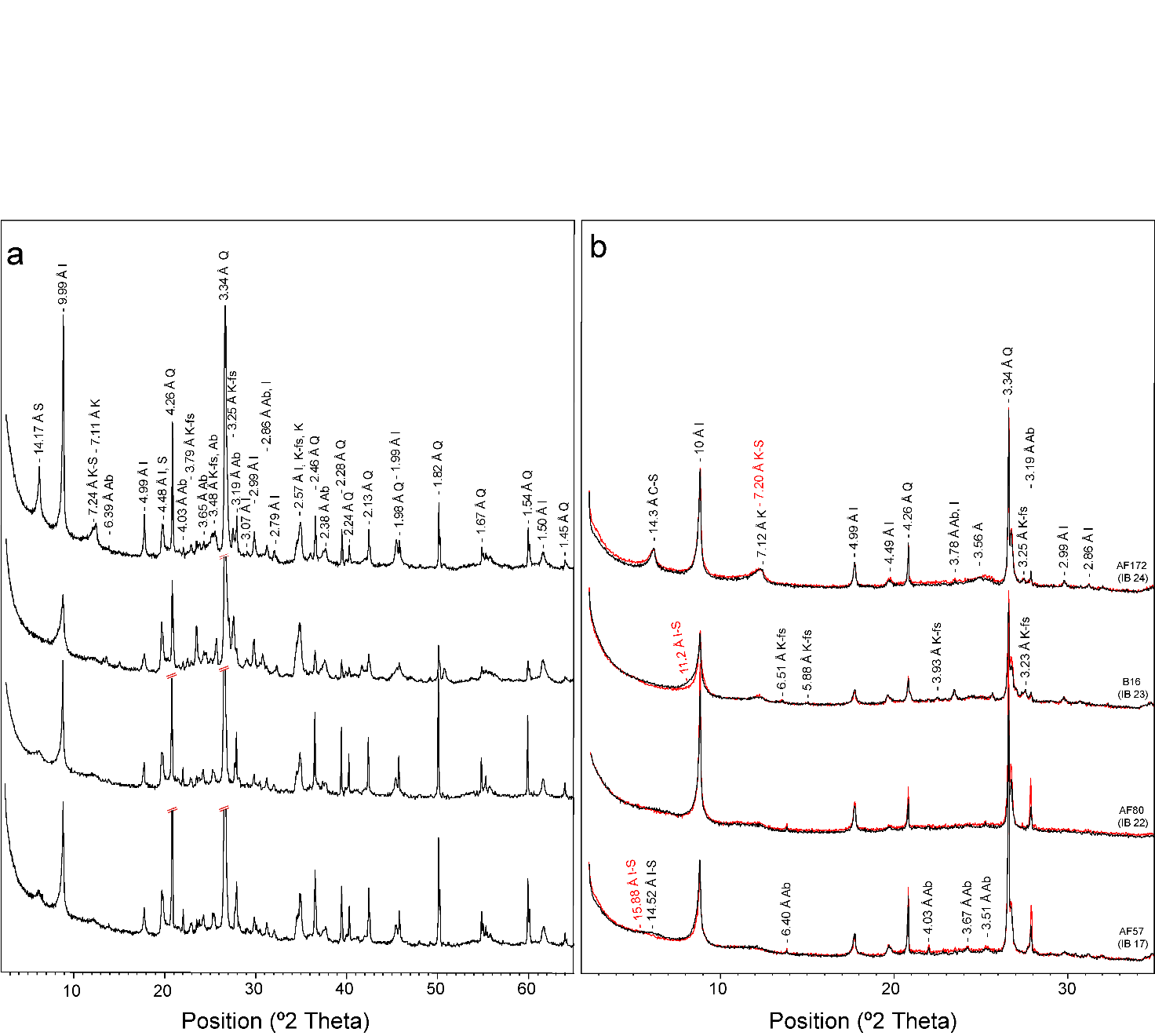


**Supplementary Figure S10. XRD patterns of representative samples from the depositional sequence 3 (distal settings of AF57; B16) and 4 (proximal settings of AF172). (a)** bulk powder and (**b)** clay fractions (< 2 µm). Sample B16 represents a fossiliferous outcrop. Note the presence of illite-rich I–S MLMs in B16. I – Illite; S – Smectite; Q – Quartz; K-fs – K-feldspar; Ab – Albite; K – Kaolinite; I-S – Illite-Smectite mixed layer; C-S – Chlorite-Smectite mixed layer; K-S – Kaolinite-Smectite mixed layer.


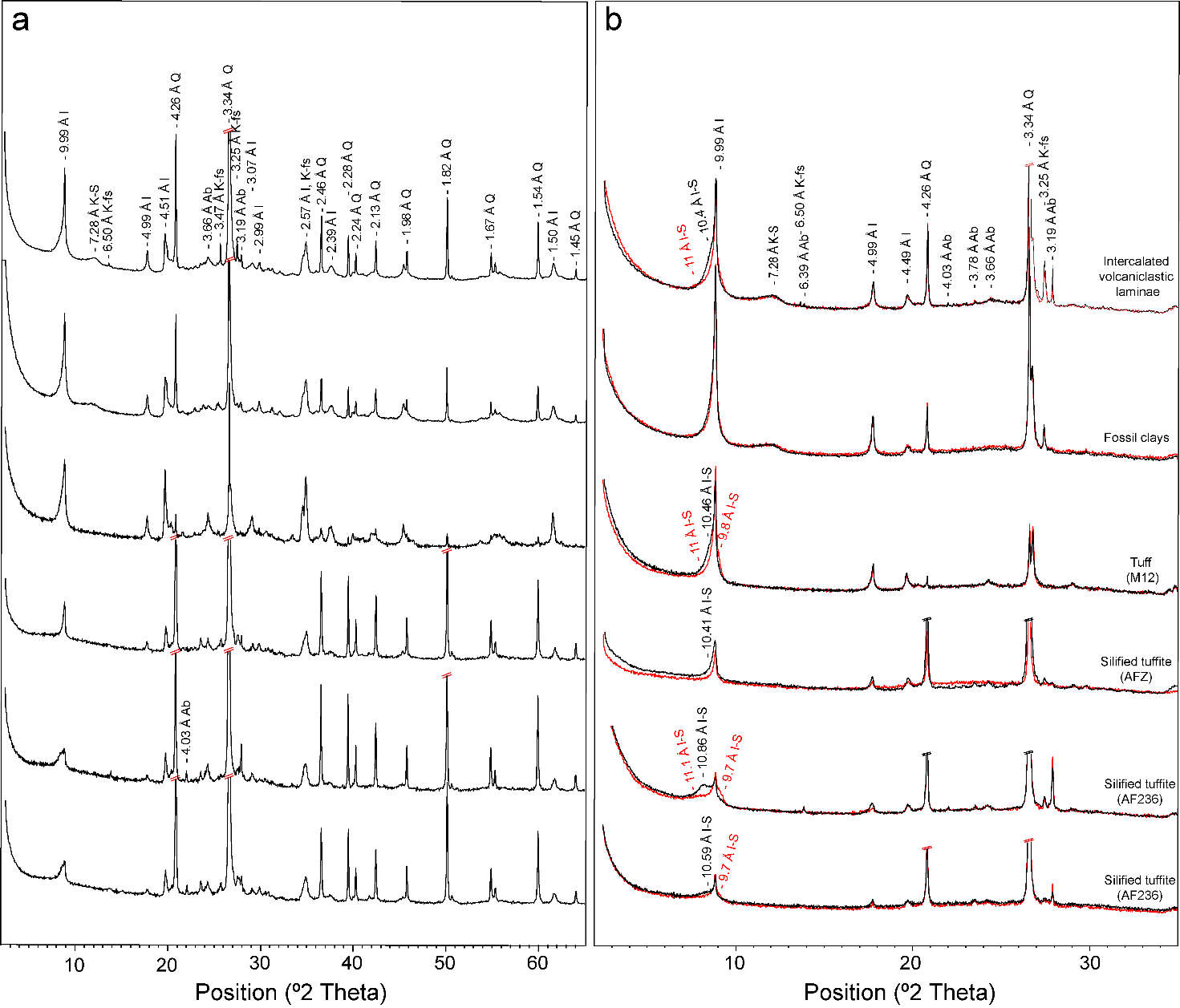


**Supplementary Figure S11. XRD patterns from the tuff (M12), tuffites (AF236, AFZ), fossil clays, and intercalated volcaniclastic coarse-grained laminae (VL). (a)** bulk powder and **(b)** clay fractions (< 2 µm). Note illite-rich I–S MLMs in all samples except those extracted from the fossil lamina. I – Illite; S – Smectite; Q – Quartz; K-fs – K-feldspar; Ab – Albite; K – Kaolinite; I-S – Illite-Smectite mixed layer; C-S – Chlorite-Smectite mixed layer; K-S – Kaolinite-Smectite mixed layer.


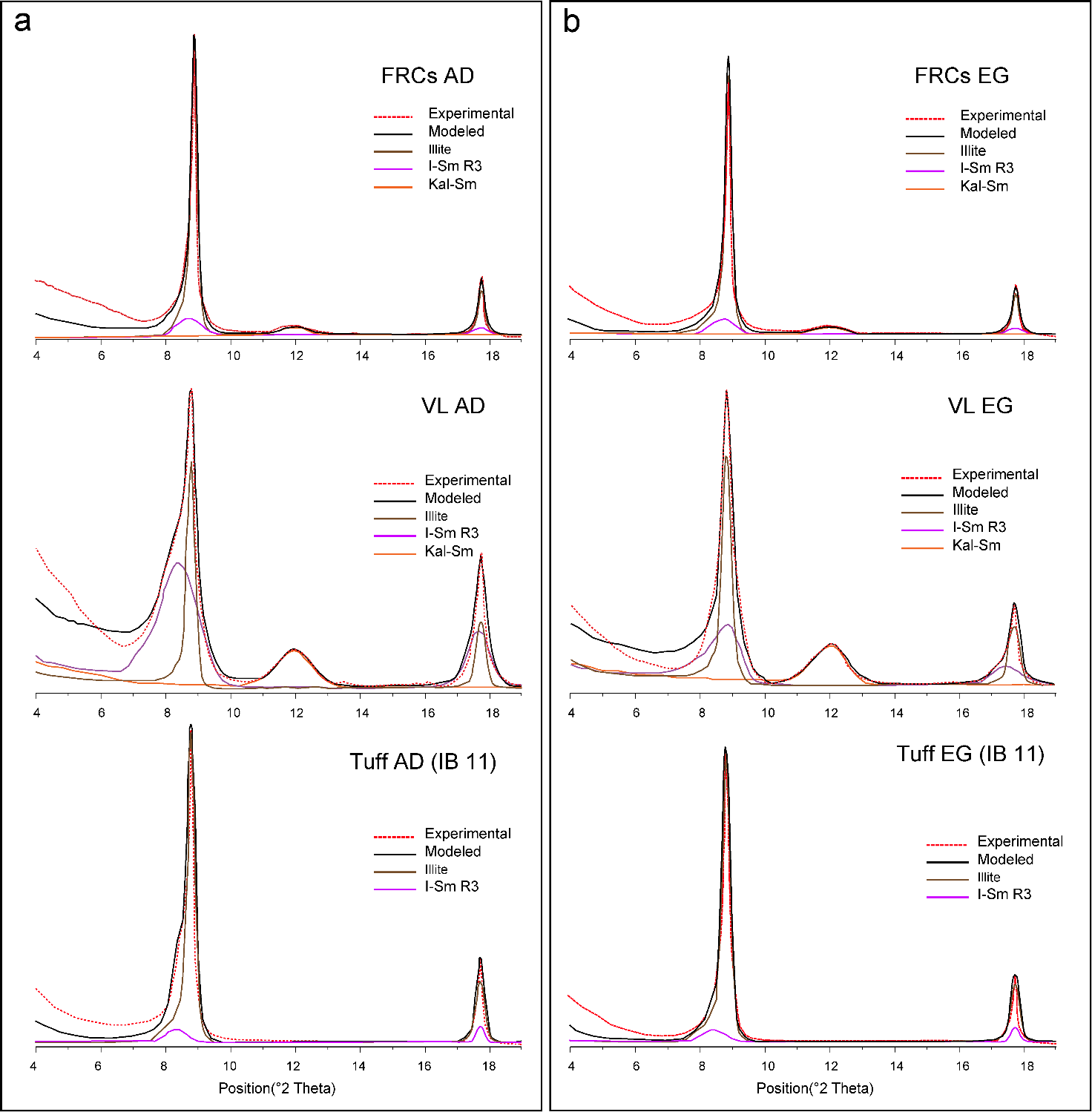


**Supplementary Figure S12. Experimental (dashed lines) and modeled (continuous lines) XRD profiles by Newmod of fossil clays (FBCs), volcaniclastic laminae (VL) and tuff clays, showing the R3 I–S MLMs ordering.** **(a)** After air-dried preparation (AD) and **(b)** after ethylene-glycol saturation (EG).


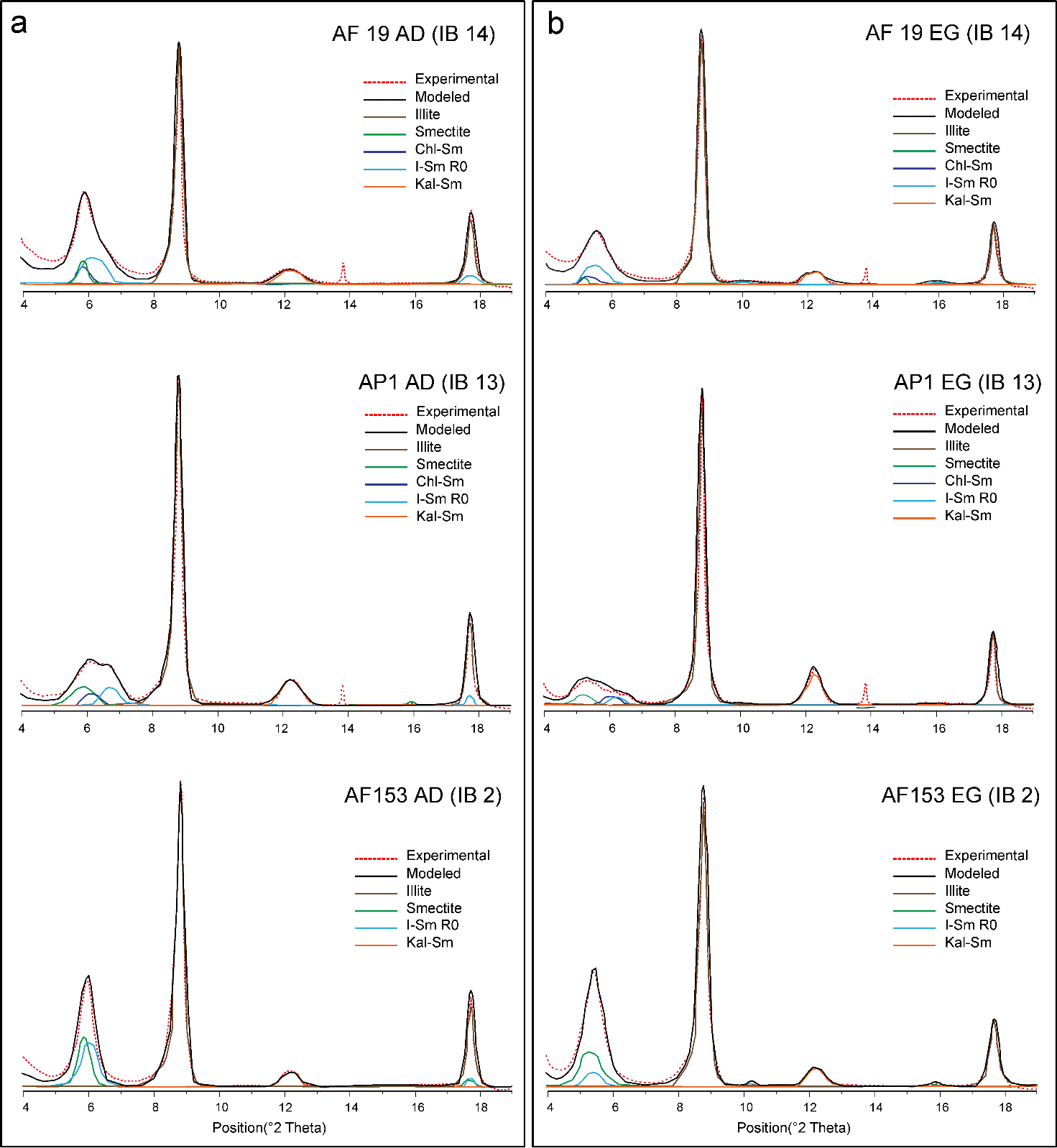


**Supplementary Figure S13. Experimental (dashed lines) and modeled (continuous lines) XRD profiles by Newmod of representative samples from smectite-rich facies, showing the R0 I–S MLMs ordering.** **(a)** After air-dried preparation (AD) and **(b)** **after** ethylene-glycol saturation (EG).


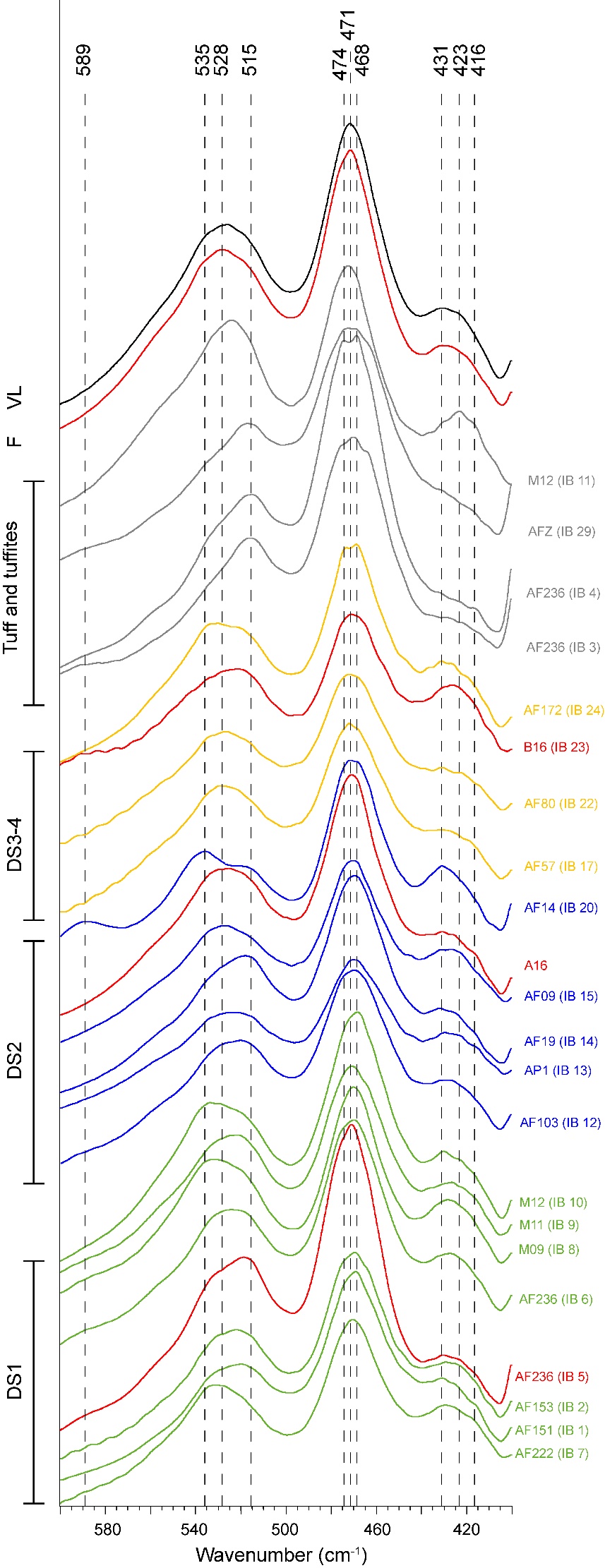


**Supplementary Figure S14. Infrared spectra of the clay fractions in the 600-400 cm^-1^ region**. The bands between 420 and 431 cm^-1^ are a mixture of the bands of illite (ca. 426 cm^-1^) and kaolinite (ca. 431 cm^-1^). The band near 470 cm^-1^ can be assigned to the Si–O–Si bending, and shifts to higher frequencies (471–472 cm^-1^) can be also related to illite or kaolinite bands, while lower frequencies (469 cm^-1^) are more similar to some reported values of smectite^12^. The bands between 515 cm^-1^ and 535 cm^-1^ can be assigned to the Si–O–Al^VI^ bending vibration^13^. Higher frequencies (~532 cm^-1^) could be related to kaolinite bands, while intermediate (~529 cm^-1^) and lower frequencies (~520 cm^-1^) are closer to illite and smectite values, respectively. Bands around ~589 cm^-1^ are attributed to albite^12^. DS1 = Depositional Sequence 1; DS2 = Depositional Sequence 2; DS3-4 = Depositional Sequence 3 and 4; F = Fossil-bearing clays; VL = intercalated coarse-grained volcaniclastic laminae. For details see Dataset S1. Image created using OMNIC 9.9.473 (www.thermoscientific.com/pm_molspec).


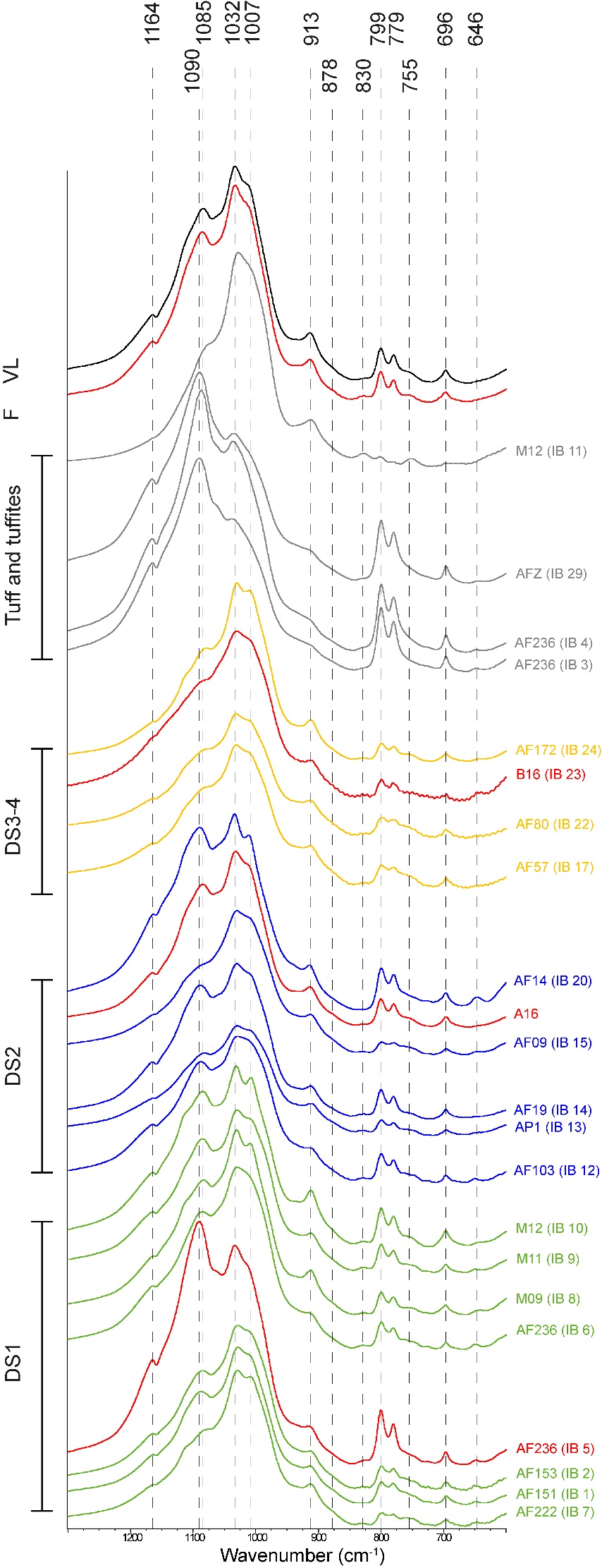


**Supplementary Figure S15**. **Infrared spectra of the clay fractions in the 1300–600 cm^-1^ region.** The broadening of the band near 1028 cm^-1^ can be attributed to the Si–O–Si stretching vibrations in samples with higher smectite content. In samples with a higher illite composition, the Si–O–Si stretching vibration appear towards higher frequencies (1032 cm^-1^), and the two strong bands near 1031 cm^-1^ and 1008 cm^-1^ can be attributed to the kaolinite Si–O–Si stretching vibrations. A weak band near 830 cm^-1^ can be assigned to the Al^IV^–O out of plane vibration or AlMgOH bending vibrations of illite^13,14^, and the band near 913 cm^-1^ to the AlOHAl bending of illite, smectite or kaolinite. A weak inflexion near 878 cm^-1^ can be related to the δ(AlFe^3+^OH) vibrations in kaolinite^15,16^. The typical absorption bands of quartz appear at 779 cm^-1^, 799 cm^-1^, and near 1085 cm^-1^ and 1163 cm^-1^. DS1 = Depositional Sequence 1; DS2 = Depositional Sequence 2; DS3-4 = Depositional Sequence 3 and 4; F = Fossil-bearing clays; VL = intercalated coarse-grained volcaniclastic laminae. For details see Dataset S1. Image created using OMNIC 9.9.473 (www.thermoscientific.com/pm_molspec).


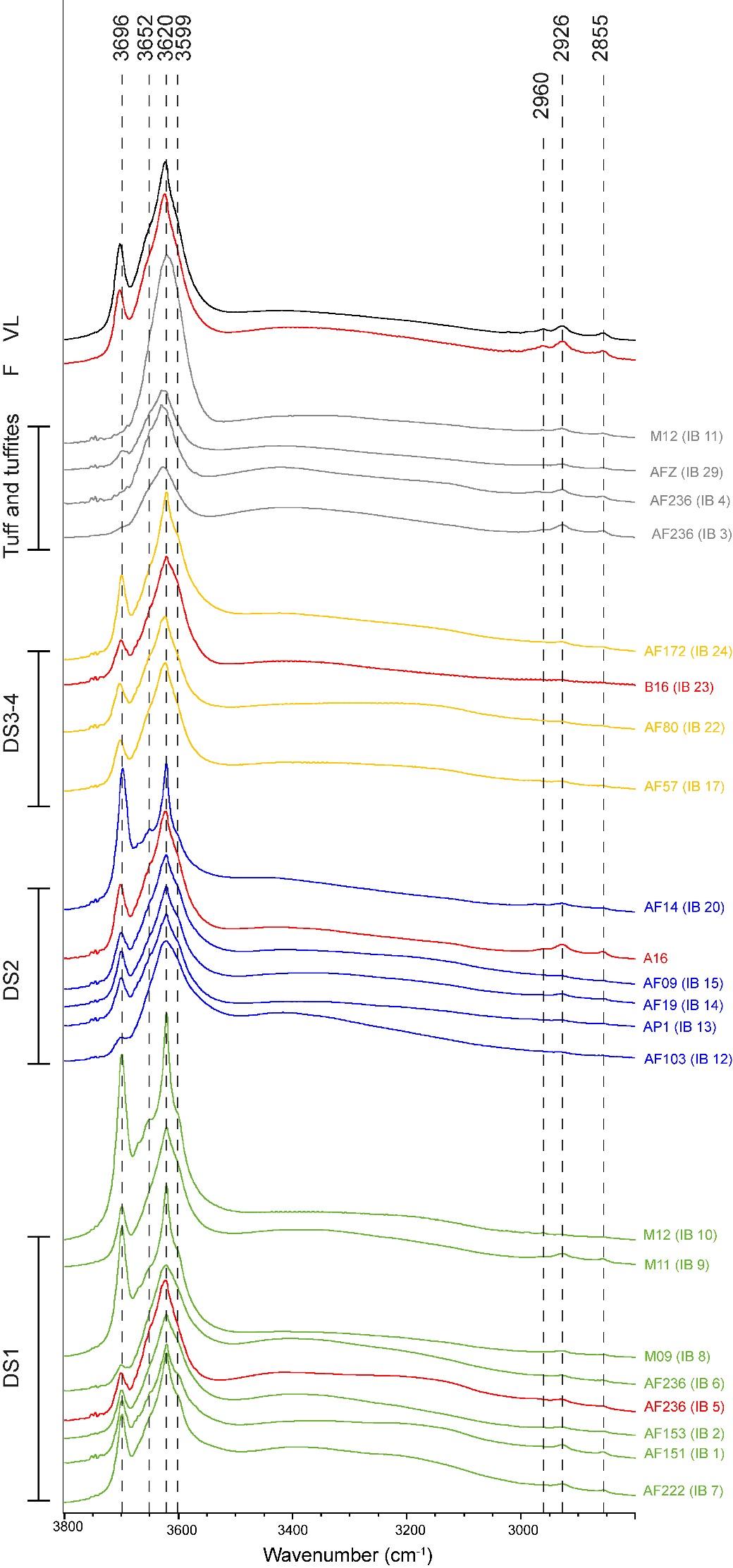


**Supplementary Figure S16**. **Infrared spectra of the clay fractions in the OH stretching region.** Absorption bands at 3696 cm^-1^, 3620 cm^-1^, ca. 3669 cm^-1^ and ca. 3650 cm^-1^ are typical of disordered kaolinite, and shoulders near 3600 cm^-1^ can be attributed to Fe^3+^ in their octahedral sheets (νAlFeOH). The broadening of the band near 3620 cm^-1^ can be related to the presence of the OH-stretching of montmorillonite in the samples higher in smectite content, as seen by XRD patterns. The broadening at the same region also can be related to illite in the illite-rich samples. The broad band near 3420 cm^-1^ in the smectite-rich samples can be related to the OH stretching of absorbed H_2_O, which were not released upon heating. Absorption bands between 3000–2800 cm^-1^ reveal the presence of aliphatic hydrocarbons, relatively more intense in the fossiliferous substrates and volcanogenic sediments, but small peaks are present throughout almost all examined samples. These bands can be assigned to symmetric CH_3_ stretching (near 2960 cm^-1^), asymmetrical CH_2_ stretching (near 2926 cm^-1^) and symmetrical CH_2_ stretching (near 2855 cm^-1^). DS1 = Depositional Sequence 1; DS2 = Depositional Sequence 2; DS3-4 = Depositional Sequence 3 and 4; F = Fossil-bearing clays; VL = intercalated coarse-grained volcaniclastic laminae. For details see Dataset S1. Image created using OMNIC 9.9.473 (www.thermoscientific.com/pm_molspec).


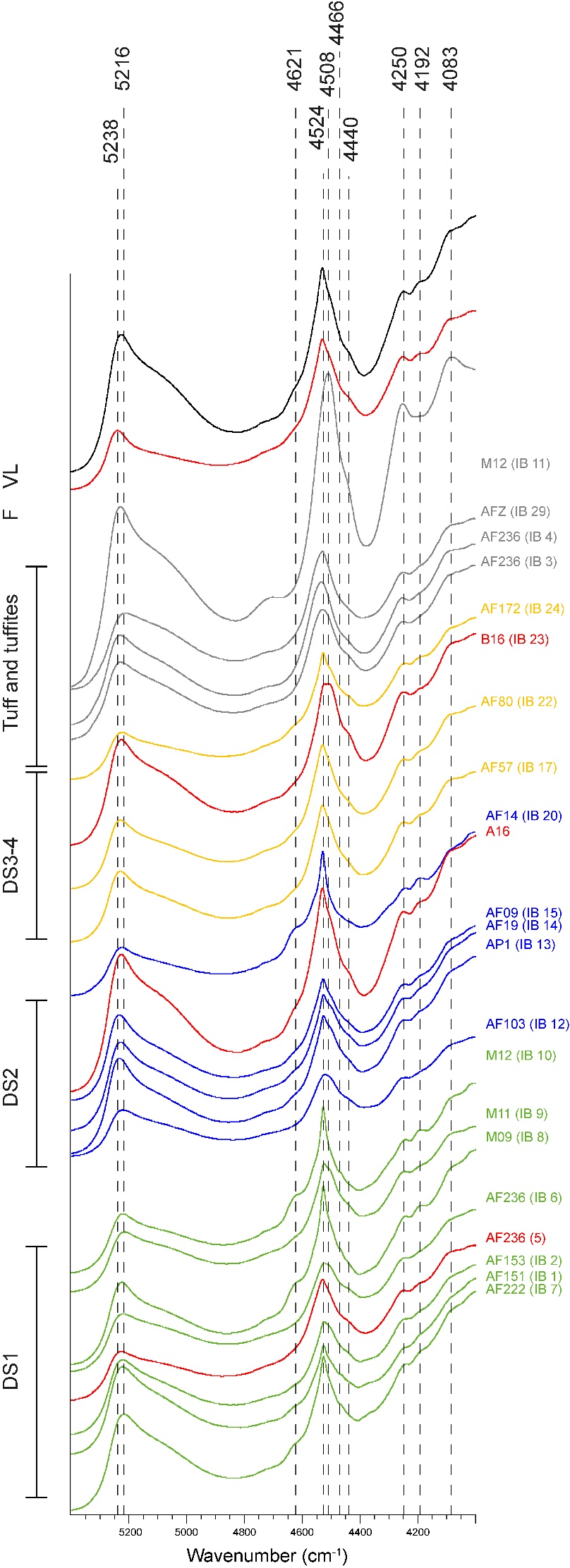


**Supplementary Figure S17**. **Near-Infrared spectra of the clay fractions in the 5300–4100 cm^-1^ region.** The narrow band near 4527 cm^-1^ can be attributed to kaolinite, as well as the weaker band near 4621 cm^-1^. The broadening of the region near 4524 cm^-1^ is observed in the smectite-rich samples, and can represent the combination bands of montmorillonite. The shoulder near 4466 cm^-1^ can be correlated with the absorption band of AlFe^3+^OH and the shoulder near 4440 cm^-1^ to (ν+δ) AlMgOH^17^. Interestingly, the last shoulder is more pronounced in the fossiliferous samples and volcanogenic sediments, corroborating the previous presence of montmorillonite, which was later illitized. The shoulder near 4500 cm^-1^ could be related to the structural OH in illite. The broad band near 5220 cm^-1^ is assigned to the combination modes of the vibrations of water molecules^17^. DS1 = Depositional Sequence 1; DS2 = Depositional Sequence 2; DS3-4 = Depositional Sequence 3 and 4; F = Fossil-bearing clays; VL = intercalated coarse-grained volcaniclastic laminae. For details see Dataset S1. Image created using OMNIC 9.9.473 (www.thermoscientific.com/pm_molspec).


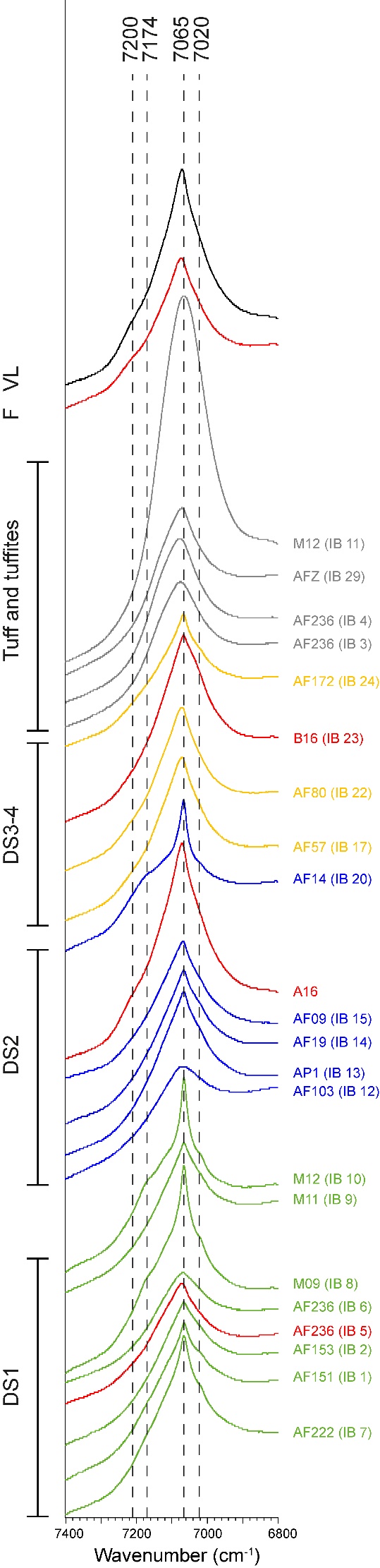


**Supplementary Figure S18**. **Near-Infrared spectra of the clay fractions in the first overtone region (7400–6800 cm^-1^).** The narrow band at 7065 cm^-1^ is attributed to the 2νAl_2_OH overtone of the OH stretching modes (3620 cm^-1^) of kaolinite^18^, and the shoulder near 7170 cm^-1^ corresponds to the first overtone of the 3669 cm^-1^ band of kaolinite^17^. Changes to higher positions near 7071 cm^-1^ likely reflects the 2ν(OH) overtone of illite, present in most of the fossiliferous samples and tuffs. The shoulder near 7020 cm^-1^ could be related to the overtone of the band at 3598 cm^-1^ (νAlOHFe^3+^) of kaolinites with Fe^3+^ substitutions. Indeed, the presence of this shoulder seems to correlate with the presence of kaolinite. DS1 = Depositional Sequence 1; DS2 = Depositional Sequence 2; DS3-4 = Depositional Sequence 3 and 4; F = Fossil-bearing clays; VL = intercalated coarse-grained volcaniclastic laminae. For details see Dataset S1. Image created using OMNIC 9.9.473 (www.thermoscientific.com/pm_molspec).

**
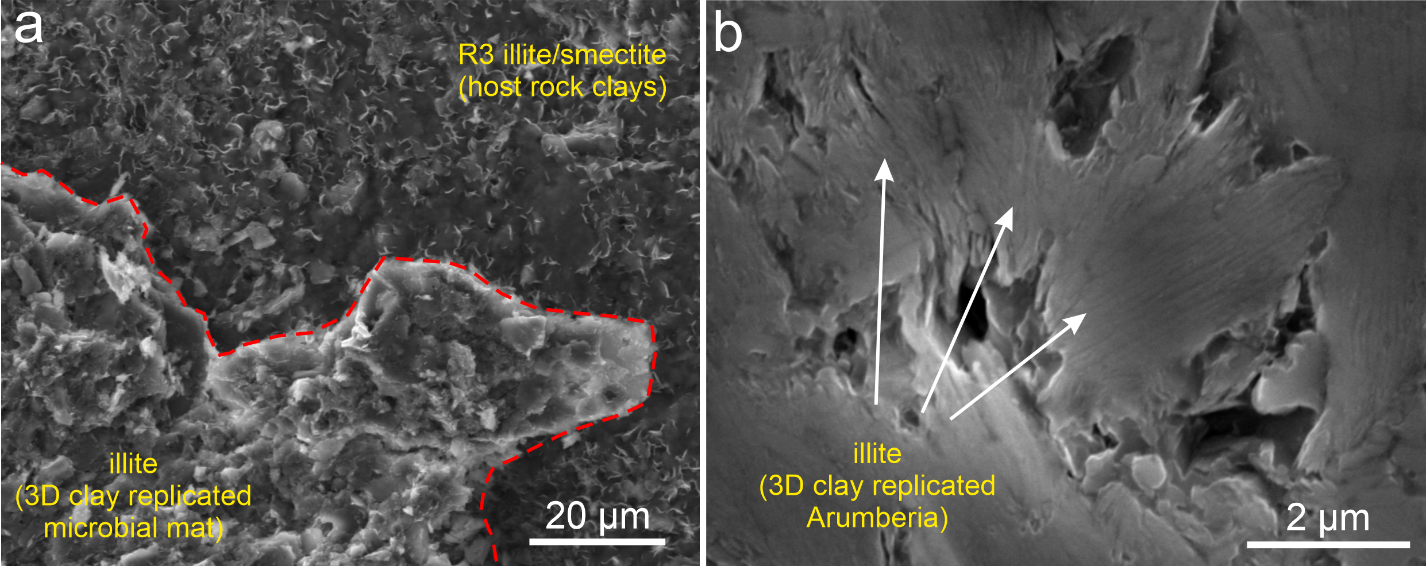
**

**Supplementary Figure S19. SEM micrographs of clay-replicated microbial mats. (a)** Interface between 3D replicated microbial tuft with the illite/smectite rich host rock. **(b)** Close-up of the illite crystals preserving Arumberia-like structures.


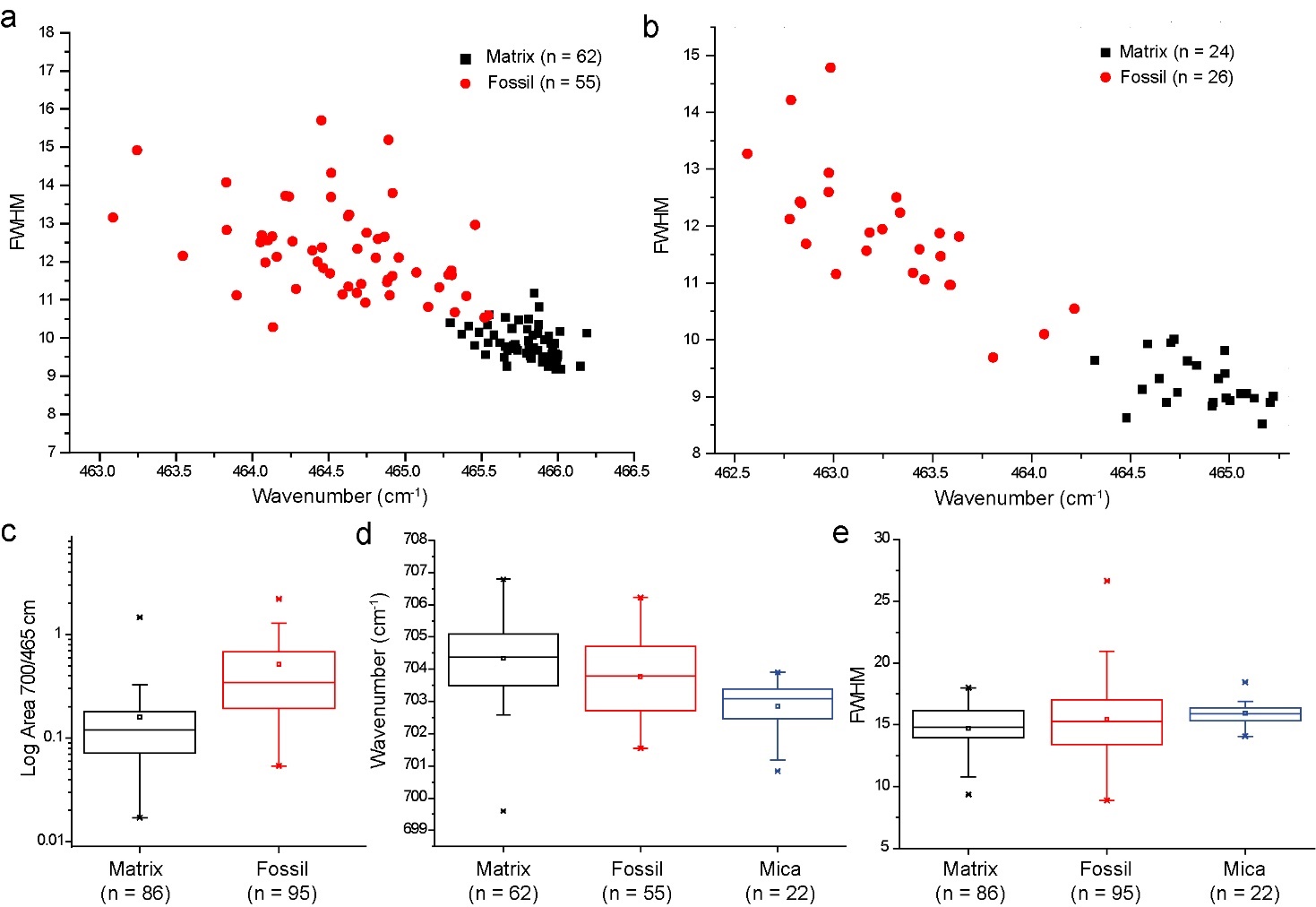


**Supplementary Figure S20. Statistical comparison of Raman fitting results from fossil clays (red), matrix clays (black) and detrital mica (blue).** **(a)** Scatter plot showing the relationship between full width at half maximum and peak position of fossil and matrix clays. Note that fossil clays present higher FWHM values and lower peak positions, due to the presence of bands of illite (and sometimes quartz) in this region. **(b)** Same as (a), but results from investigations realized in a different day. **(c)** Box plot of the 700/465 cm^-1^ area ratio, showing higher proportion of area of the peak near 700 cm^-1^ (characteristic of phyllosilicates) in the fossils. **(d)** Box plot of the position of the peak near 700 cm^-1^ for the clays in the matrix and the fossils, as well as for micas. Note similar values for fossils and matrix clays. **(e)** FWHM of the peak near 700 cm^-1^ for each type of clay. Note the higher range of FWHM in the spectra from the fossil clays due to lower degrees of crystallinity. On the other hand, detrital micas show lower values of FWHM suggesting higher crystallinity.


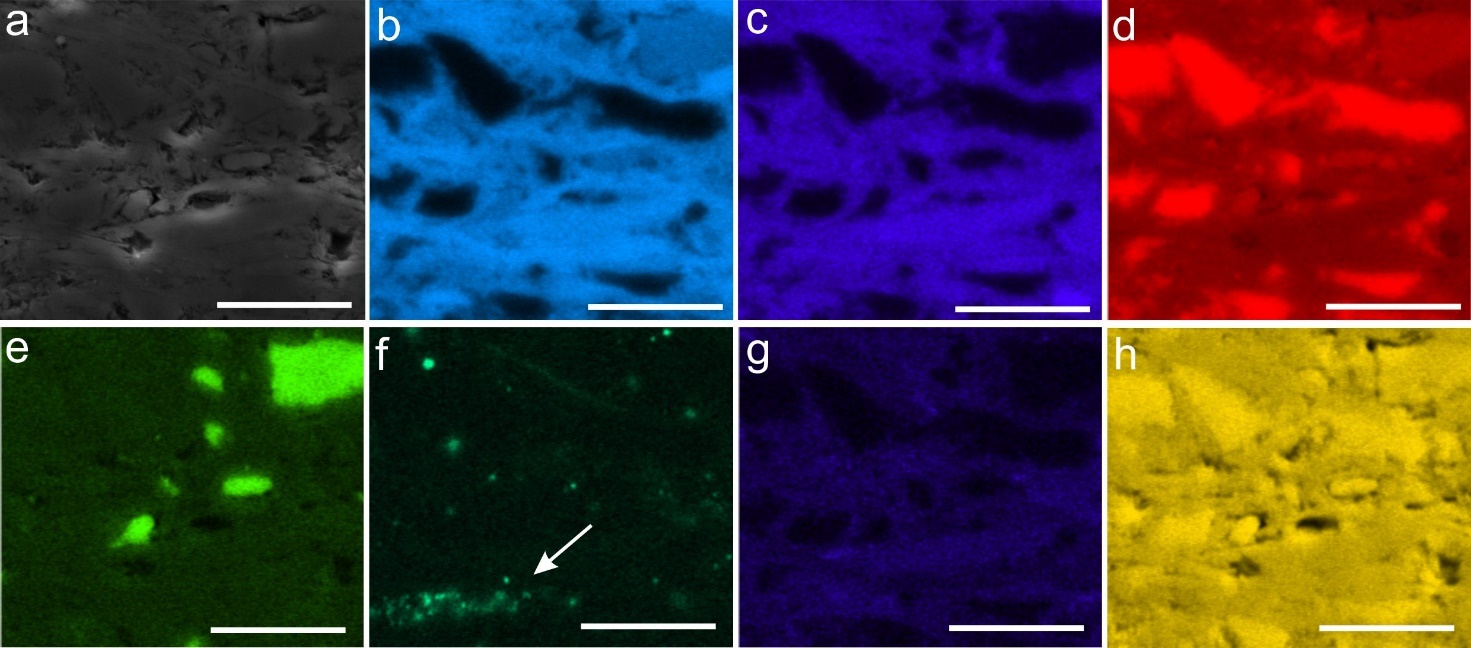


**Supplementary Figure S21. EDS maps of a small region inside a three-dimensionally preserved Arumberia-type microbial mat. (a)** electron image. Elemental maps of **(b)** aluminum, **(c)** potassium, **(d)** silicon, **(e)** sodium, **(f)** titanium, highlighting fine-grained anatase concentrations (e.g. arrow), **(g)** iron, and **(h)** oxygen. Scale: 10 µm. Image created using INCA (a, c; <https://www.etas.com/en/products/inca_software_products.php>).


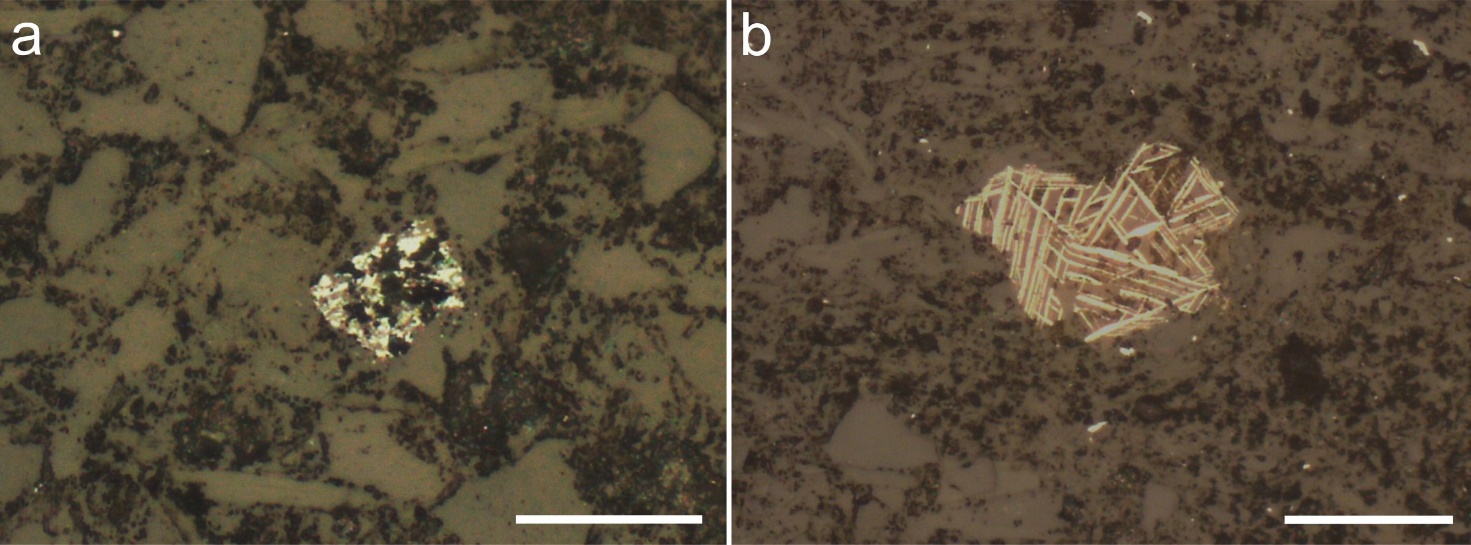


**Supplementary Figure S22. Leucoxene grains**. **(a)** Leucoxene after Fe-Ti oxide mineral (probably ilmenite). **(b)** Leucoxene after titanomagnetite, presenting characteristic trellis texture. Scales: 50 µm.

**Movie S1 (separate file).** Microtomographic movie composed of horizontal sections (bedding plane) of a sample with 3D clay mineralized microbial tufts.

**Dataset S1 (separate file).** Infrared absorption bands of the selected samples (clay fraction)

**Dataset S2 (separate file).** Oxide wt% results and calculated structural formula of illite crystals from fossils

**Dataset S3 (separate file).** Raman fitting results for the fossil and cement/matrix (C/M) clay minerals, and detrital micas

**Dataset S4 (separate file).** Raman spectroscopy polymorph identification in randomly chosen TiO2 crystals from fossils (3D Arumberia mats) and sedimentary matrix

**SI References**

1. Rostirolla, S. P., Alkmim, F. F. & Soares, P. C. O Grupo Itajaí, Estado de Santa Catarina, Brasil: Exemplo de sedimentação em uma bacia flexural de antepaís. *B. Geoci. PETROBRÁS* **6**, 109–122 (1992).

2. Rostirolla, S. P., Ahrendt, A., Soares, P. C. & Carmignani, L. Basin analysis and mineral endowment of the Proterozoic Itajai Basin, south-east Brazil. *Basin Res.* **11**, 127–142 (1999).

3. Rostirolla, S. P. Tectônica e Sedimentação da Bacia do Itajaí – SC. (UFOP, 1991).

4. Basei, M. A. S. *et al.* The Itajaí foreland basin: A tectono-sedimentary record of the Ediacaran period, Southern Brazil. *Int. J. Earth Sci.* **100**, 543–569 (2011).

5. Guadagnin, F. *et al.* Depositional age and provenance of the Itajaí Basin, Santa Catarina State, Brazil: Implications for SW Gondwana correlation. *Precambrian Res.* **180**, 156–182 (2010).

6. Becker-Kerber, B. *et al.* The oldest record of Ediacaran macrofossils in Gondwana (~563 Ma, Itajaí Basin, Brazil). *Gondwana Res.* **84**, 211–228 (2020).

7. Gresse, P. G., Chemale, F., da Silva, L. C., Walraven, F. & Hartmann, L. A. Late- to post-orogenic basins of the Pan-African - Brasiliano collision orogen in southern Africa and southern Brazil. *Basin Res.* **8**, 157–171 (1996).

8. Fonseca, M. M. Sistemas deposicionais e estratigrafia de sequências da Bacia do Itajaí, SC e detalhamento do Complexo Turbidítico de Apiúna. (UNISINOS, 2004).

9. Teixeira, A. L., Gaucher, C., Paim, P. S. G., Fonseca, M. M. & Filho, W. F. S. Bacias do estágio de Transição da Plataforma Sul-Americana. in *Geologia do Continente Sul-Americano: Evolução da Obra de Fernando Flávio de Almeida* (eds. Mantesso-Neto, V., Bartorelli, A., Carneiro, C., Dal, R. & Brito Neves, B. B.) 487–536 (Beca Produções Culturais Ltda, 2004).

10. Weaver, C. & Pollard, L. *The Chemistry of Clay Minerals*. (Elsevier Science, 1973).

11. Meunier, A. *Clays*. *Clays* (Springer-Verlag, 2005). doi:10.1007/b138672.

12. Russell, J. D. & Fraser, A. R. Infrared methods. in *Clay Mineralogy: Spectroscopic and Chemical Determinative Methods* (ed. Wilson, M. J.) 11–67 (Springer Netherlands, 1994). doi:10.1007/978-94-011-0727-3_2.

13. Farmer, V. C. *The Infrared Spectra of Minerals*. (Mineralogical Society of Great Britain and Ireland, 1974). doi:10.1180/mono-4.

14. Zviagina, B. B., Drits, V. A., Środon´, J., McCarty, D. K. & Dorzhieva, O. V. The illite aluminoceladonite series: Distinguishing features and identification criteria from X-ray diffraction and infrared spectroscopy data. *Clays Clay Miner.* **63**, 378–394 (2015).

15. Mendelovici, E., Yariv, S. & Villalba, R. Iron-bearing kaolinite in Venezuelan laterites: I. Infrared spectroscopy and chemical dissolution evidence. *Clay Miner.* **14**, 323–331 (1979).

16. Petit, S. & Decarreau, A. Hydrothermal (200°C) synthesis and crystal chemistry of iron-rich kaolinites. *Clay Miner.* **25**, 181–196 (1990).

17. Madejová, J., Gates, W. P. & Petit, S. *IR Spectra of Clay Minerals*. *Developments in Clay Science* vol. 8 (2017).

18. Petit, S., Madejová, J., Decarreau, A. & Martin, F. Characterization of octahedral substitutions in kaolinites using near infrared spectroscopy. *Clays Clay Miner.* **47**, 103–108 (1999).
